# Effects of UV-C Combined with Different Antioxidants on Storage Quality and Flavor of Selenium-Sand Melon Juice

**DOI:** 10.3390/foods15091485

**Published:** 2026-04-24

**Authors:** Li-Li Li, Meng-Yao Fan, Zhi-Jing Ni, Run-Hui Ma, Zhao-Jun Wei, Kiran Thakur

**Affiliations:** 1 School of Biological Science and Engineering, North Minzu University, Yinchuan 750021, China; lililyplus@163.com (L.-L.L.); lovebear@vip.163.com (Z.-J.N.); zhantingbaiyang@163.com (R.-H.M.); zjwei@hfut.edu.cn (Z.-J.W.); 2 Ningxia Key Laboratory of Development and Utilization of Specialty Food Resources, Yinchuan 750021, China; 3 School of Food and Biological Engineering, Hefei University of Technology, Hefei 230009, China; 15555122657@163.com

**Keywords:** UV-C, inactivation, antioxidant hurdle, selenium-sand melon juice, storage, quality

## Abstract

Selenium-sand melon (*Cucumis melo* L.) juice (SSJ) is valued for its lycopene and organic selenium content, but its shelf-life is limited by heat-labile nutrients and postharvest microbial spoilage. Non-thermal strategies that combine UV-C with natural antioxidants are therefore of interest. This study quantified the individual and interactive effects of UV-C alone or with four antioxidant systems on microbial safety, bioactive retention, and the flavor stability of SSJ under extreme contamination conditions (*Escherichia coli* D25015 at 5.19 log_10_ CFU/mL; *Mucor circinelloides* D11624 at 4.36 log_10_ CFU/mL). For this, we evaluated the efficacy of five treatments: UV-C alone (Group Z) and UV-C combined with catechin (Group EC, 0.01%), sodium erythorbate (Group K, 0.01%), ascorbic acid (Group VC, 0.1%), and catechin-ascorbic acid (Group HH, 0.005% + 0.05%). Conventional pasteurization (high-temperature short-time, HTST; low-temperature long-time, LTLT) served as controls. UV-C alone (Group Z) preserved lycopene and volatile flavor compounds better than HTST or LTLT. The combined use of UV-C and antioxidants exhibited synergistic effects, with no viable bacteria detected in Group K (sodium erythorbate) within four weeks. UV-C combined with antioxidants offer a scalable, non-thermal strategy that maintains nutritional and sensory quality while achieving pathogen reduction. These findings provide a quantitative framework for clean-label preservation of functional melon beverages.

## 1. Introduction

Selenium-enriched sand melon (*Cucumis melo* L. var. *saccharinus*) (SSM) accumulates 20–40 mg Se kg^−1^ DW and 25–70 mg lycopene 100 g^−1^ FW, making its juice (SSJ) a high-value functional beverage [1]. However, SSJ has a high initial microbial load (typically 5–6 log CFU mL^−1^) and is highly prone to microbial spoilage. Its pigments are sensitive to oxidation, light, and enzymatic reactions, leading to rapid degradation. Moreover, the high reducing sugar content and low acidity (pH 5.6–6.1) create favorable conditions for microbial growth, further accelerating spoilage [2].

SSM contains a wide array of volatile compounds; its characteristic fragrance arises from a complex mixture of aldehydes, esters, alcohols, ketones, and hydrocarbons [3]. Conventional thermal pasteurization, such as high-temperature short-time (HTST) or low-temperature long-time (LTLT), reliably reduces pathogens but also induces thermal cis-isomerization of lycopene (30–45% loss) and generates Strecker aldehydes and furans responsible for cooked off-notes [3]. For example, thermal processing of watermelon juice produces characteristic cooked off-flavors, with octanol, diisopropyl disulfide, and (E)-2-decenal identified as major contributors to undesirable sulfurous, metallic, and fatty notes [4].

On the other hand, non-thermal technologies offer promising alternatives. A 2025 meta-analysis of over 60 juice studies confirmed that UV-C combined with mild heat (≈50 °C), pulsed electric fields (PEF), ultrasound, or natural antimicrobials reproducibly achieved ≥5-log pathogen reduction, extended shelf-life by 1–4 weeks, and better retained color, phenolics, and fresh aroma compared to thermal pasteurizations [5]. Another recent meta-analysis showed that cold-plasma and PEF treatments increase d-limonene and E-2-hexenal levels in melon-type juices, whereas HTST persistently depletes these markers [6], emphasizing the advantage of non-thermal UV-C-based strategies for fruit juices [7].

Ultraviolet-C (UV-C, λ max 254 nm) is an FDA-approved non-thermal alternative that forms pyrimidine dimers and blocks microbial DNA replication without leaving chemical residues. UV-C (390 mJ cm^−2^) combined with mild heat (50 °C) delivered ≥5-log kill of *E. coli, L. plantarum* and *S. cerevisiae* in pilot-scale turbid juices, with refrigerated storage providing an additional hurdle against bacterial recovery [8]. Similarly, a hurdle approach combining UV-C (1.27 kJ cm^−2^), mild heat (50 °C), and 30 ppm natamycin achieved ≥5-log kill of *E. coli*, Salmonella, and yeasts in apple juice, prevented microbial regrowth for 23 d at 7 °C, and retained consumer-acceptable sensory and nutritional quality [9].

Combining UV-C with food-grade antioxidants can scavenge UV-induced reactive oxygen species (ROS), decrease the required UV dose, and preserve heat-labile micronutrients. A recent study showed that a binary system of 0.005% (+)-catechin-0.05% ascorbic acid system (HH) inhibited lycopene cis-isomerization by 72% compared with UV-C alone [9]. The HH system also provides synergistic metal-chelating and radical-scavenging capacity [3].

Despite these advances, the interactive impact of UV-C combined with antioxidant systems on selenium speciation, lycopene retention, and key odorants in SSJ have not been quantified under controlled contamination conditions. Therefore, the objective of this study was to develop a predictive, non-thermal preservation matrix for SSJ by evaluating the microbial efficacy and quality kinetics of UV-C combined with catechin, sodium erythorbate, ascorbic acid or the HH system, compared with traditional thermal methods (HTST and LTLT).

## 2. Materials and Methods

### 2.1. Reagents

Microbial strains were purchased from the Shanghai Bioresource Collection Center. Culture media, including LB broth, Potato Dextrose Agar (PDA) and Brain Heart Infusion (BHI) agar, were obtained from Hobio Biotechnology Co., Ltd. (Qingdao, China). Anhydrous ethanol, potassium dihydrogen phosphate, and dipotassium hydrogen phosphate were supplied by Shanghai Yuan’an Biological Technology Co., Ltd. (Shanghai, China). All chemical reagents were of analytical grade. The internal standard, 1,2-dichlorobenzene (internal standard, ≥99.5%), was obtained from Tanmo Quality Inspection Technology Co., Ltd. Antioxidant compounds (catechin, sodium erythorbate, and ascorbic acid) were purchased from Shaanxi Ronglin Biotechnology Co., Ltd., Xi’an, China.

### 2.2. SSJ Sample Preparation and Processing

#### 2.2.1. Preparation of SSJ

The experimental raw material, SSM (Golden City 5 variety), was purchased from a local market in Ningxia, China. The fruits were washed, destemmed, and chopped into small pieces. Juice was extracted using a Joyoung Z5-LZ550 juicer (120 W, Joyoung Co., Ltd., Jinan, China) and filtered through an 80-mesh sieve. The juice was stored at 4 °C until further use [2].

#### 2.2.2. UV-C254 nm Equipment

A custom-built liquid food UV-C decontamination system (developed by Professor Chen Jun’s laboratory at Nanchang University) was used for UV-C treatment. The system operated in a recirculation mode, with juice pumped through UV-C-transparent tubing at a controlled rate. The UV-C lamp (254 nm) had a total output power of 140 W. Strawberry juice inoculated with target microorganisms was pumped through the reactor at a flow rate of 300 mL/min using a peristaltic pump. The process was repeated six times. The calculated UV-C irradiation dose was 168 J/mL, based on the following formula:

(1) $$\mathrm{UV}\text{-}C\;\mathrm{dose}\;\left(J/\mathrm{mL}\right)=\frac{\mathrm{Total}\;\mathrm{UV}\text{-}C\;\mathrm{output}\;\mathrm{power}\;(W)}{\mathrm{Flow}\;\mathrm{rate}\;(\mathrm{mL}/s)}$$

#### 2.2.3. Thermal Sterilization

Thermal treatments were performed in a 30 L laboratory-scale pasteurization tank (PA-TK-30, 380 V, 50 Hz, Shanghai Shunyi Experimental Equipment Co., Ltd., Shanghai, China) at 65 °C for 30 min (LTLT) and 85 °C for 15 s (HTST). After treatment, samples were rapidly cooled in an ice-water bath and then equilibrated to room temperature before analysis [10].

### 2.3. Microbiological Analysis

#### 2.3.1. Preparation and Inoculation of Bacterial Suspension

*Escherichia coli D25015* (*E. coli*) and *Mucor circinelloides D11624* (*M. circinelloides*), selected for their resistance in preliminary studies [11], were used as target microorganisms. To prepare the bacterial inoculum, a single *E. coli* colony was cultivated in 10 mL LB broth at 35 ± 2 °C for 12 h [12]. One milliliter of this pre-culture was transferred to 100 mL fresh LB broth and shaken at 180 rpm for additional 12 h under the same conditions. Cells were pelleted (5000 rpm, 5 min, 4 °C), washed twice with sterile PBS, and re-suspended in the same buffer. The suspension was added to SSJ at 0.2% (*v*/*v*) to yield ~10^5^ CFU mL^−1^ [13]. For the fungal inoculum, 10 mL sterile PBS was poured over a *M. circinelloides* slant, and the surface was gently scraped with a loop. The resulting suspension was filtered through an 80-mesh sieve to remove hyphae, counted in a hemocytometer, and adjusted to 10^7^ CFU mL^−1^. Spores were introduced into SSJ at 0.1% (*v*/*v*) to give a final density of ~10^4^ CFU mL^−1^ [14].

#### 2.3.2. Microbial Count

For microbial enumeration, serial dilutions of SSJ samples were prepared in sterile PBS (pH 7.4), and then inoculated onto selective media by either the drop-plate method or spread-plate method. BHI agar was used for total bacterial counts, while PDA was employed for fungal culture. Inoculated BHI agar plates were incubated at 37 °C for 24 h, and PDA plates at 28 °C for 48 h. After incubation, colonies were counted using a colony counting camera under a 5V/1A white LED light source. To ensure counting accuracy, the operation was carried out at a vertical observation angle (approximately 90° to the plate surface). All determinations were performed in triplicate; the results were averaged and expressed as colony-forming units per milliliter (CFU·mL^−1^) [15].

### 2.4. Physicochemical Characterization

#### 2.4.1. Color Measurement

The chroma value was determined by colorimeter DS- 200 in reflection mode at 25 °C, with a standard whiteboard as reference. SSJ (25 mL) was placed into a cuvette, and the CIELAB parameters (L*, a*, and b*) were measured. Results were expressed as the total color difference (ΔE) calculated as follows:

(2) $$\Delta E\;=\sqrt{{\left(L_{i}^{*}-L_{0}^{*}\right)}^{2}+{\left(a_{i}^{*}-a_{0}^{*}\right)}^{2}+{\left(b_{i}^{*}-b_{0}^{*}\right)}^{2}}$$

L* denotes brightness, a* indicates the red–green axis, and b* the yellow–blue axis; subscripts i and 0 refer to post- and pre-test values, respectively [16].

#### 2.4.2. Titratable Acidity

TA was measured by diluting 5 mL of SSJ to 100 mL with distilled water and titrating with 0.05 M NaOH to a faint pink endpoint (phenolphthalein, 30 s). Results were reported as g citric acid per 100 mL juice:

(3) $$\mathrm{TA}=100\times \frac{V_{1}\times C\times 0.064}{V_{2}}$$

where V1 denotes the volume of standard solution consumed, mL; C denotes the molar concentration of the standard solution, 0.05 mol/L; V2 denotes the volume of the sample, mL; and 0.064 denotes the conversion factor, in terms of citric acid [2].

#### 2.4.3. Soluble Solid Content

Soluble solid content was measured by a portable digital refractometer (PAL- α, ATAGO Ltd., Tokyo, Japan). After calibration with distilled water, 0.5 mL of SSJ was placed on the prism, and the reading was recorded in Brix [2].

#### 2.4.4. pH Measurement

The pH of SSJ was measured with a digital pH meter (PHS-25, Shanghai Instrument Science and Technology Co., Ltd., Shanghai, China) at 25 °C [17].

#### 2.4.5. Total Sugar and Reducing Sugar

Total and reducing sugars were determined by the 3,5-dinitrosalicylic acid (DNS) method [17]. The glucose solution was prepared to obtain the standard curve (y = 4.039x + 0.066, R2 = 0.996).

#### 2.4.6. Lycopene Content

Lycopene was extracted using a mixture of n-hexane, acetone, and anhydrous ethanol (2:1:1, *v*/*v*/*v*). Subsequently, 1 mL of SSJ was mixed with 25 mL of the solvent mixture and magnetically stirred at 4 °C for 15 min. After adding 15 mL of distilled water and stirring for an additional 5 min, the mixture was allowed to separate. The absorbance of the upper n-hexane layer was measured at 503 nm. Quantification was performed using a Sudan red standard curve (y = 0.142x + 0.070, R^2^ = 0.994).

### 2.5. Determination of Antioxidant Activity

#### 2.5.1. DPPH Free Radical Scavenging Rate

For DPPH scavenging activity [17], 1 mL of SSJ was mixed with 1.0 mL of 0.2 mmol/L DPPH ethanolic solution and incubated in the dark for 30 min. Absorbance was measured at 517 nm. A standard curve of ascorbic acid was prepared (y = 7602.005x − 10.616, R^2^ = 0.995), and results were shown as mg ascorbic acid equivalent (AAE) per 100 mL of sample [16].

#### 2.5.2. ABTS^•+^ Free Radical Scavenging Rate

ABTS^•+^ scavenging activity was quantified following previous method with minor adjustments. The radical cation was generated by combining 7 mmol L^−1^ ABTS with 2.45 mmol L^−1^ potassium persulfate (1:1, *v*/*v*) and storing the mixture in darkness at 3 °C for 16 h. Before using it, the deep-blue solution was diluted with ethanol to an absorbance of 0.700 ± 0.020 at 734 nm. SSJ (200 µL) was mixed with 800 µL of the diluted ABTS^•+^ reagent, and the absorbance was recorded after 10 min. A calibration curve constructed with ascorbic acid (y = 419.4x + 4.825, R^2^ = 0.995) served to convert inhibition values into mg ascorbic acid equivalents (AAE) per 100 mL juice [17].

### 2.6. SPME and GC-MS Analysis

Volatile organic compounds (VOCs) were profiled by SPME–GC-MS following Wang et al. [18] with slight modifications. SSJ (8 mL) was transferred to a 20 mL glass vial containing 3 g NaCl and 20 µL of 1,2-dichlorobenzene (10 µg mL^−1^) as the internal standard. The vial was preheated at 40 °C for 20 min, and VOCs were extracted using a SPME fiber for 20 min under the same temperature. Desorption was carried out in the GC injection port at 250 °C for 5 min.

GC-MS analysis was performed using an Agilent 7000D Triple Quadrupole GC/MS system (Shimadzu, Kyoto, Japan) equipped with an HP-5MS capillary column (30 m × 250 μm × 0.25 μm). Helium (1.0 mL min^−1^) served as carrier gas; the injector was held at 250 °C. The oven temperature program was as follows: initial temperature 40 °C held for 3 min, increased to 140 °C at 4 °C min^−1^ and held for 3 min, then increased to 220 °C at 8 °C min^−1^ and held for 5 min. Mass spectra were recorded in full-scan mode (*m*/*z* 35–550) and matched against the NIST 14.0 library; only matches with ≥80% similarity were accepted. Quantification relative to the internal standard gave concentrations in µg mL^−1^; all samples were analyzed in triplicate [17,19].

### 2.7. Statistical Analysis

All experiments were performed in triplicate, and results are presented as mean ± standard deviation. Analysis of variance (ANOVA) was performed using IBM SPSS Statistics 20.

## 3. Results and Discussion

### 3.1. Effect of Different Treatments on the Physicochemical Stability of SSJ During Storage

#### 3.1.1. Total Colony Counts

**Figure 1:**
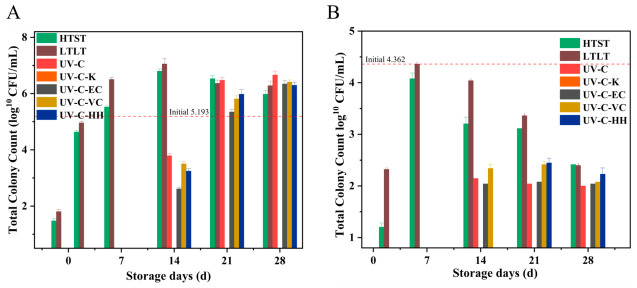
Changes in total colony counts of *Escherichia coli* (**A**) and *Mucor circinelloides* (**B**) in different treated SSJ samples during 28 d storage.

Figure 1A and B showed that the initial colony counts of *E. coli* and *M. circinelloides* in SSJ were 5.193 and 4.362 log_10_ CFU mL^−1^, respectively. After thermal treatments (HTST, 85 °C/15 s and LTLT, 65 °C/30 min), *E. coli* was reduced to 1.48 and 1.81 log_10_ CFU mL^−1^, respectively, but the mold was not eliminated. After 7 d at 4 °C, *E. coli* regrew to 5.52 (LTLT) and 6.51 (HTST) log_10_ CFU mL^−1^ levels, exceeding the initial counts. Although heat treatment effectively inactivates most microorganisms by inducing protein denaturation, the remaining heat-resistant strains can recover and regrow during refrigerated storage, resulting in the observed microbial rebound [20]. In contrast, UV-C irradiation combined with antioxidants delayed or suppressed microbial regrowth during the 4-week storage period, with varying efficacy among treatments. Notably, only Group K achieved complete suppression, with no colonies detected throughout the entire 28-day observation period. Group HH suppressed *M. circinelloides* regrowth until day 14, while other UV-C combinations showed regrowth beginning on day 7. The ranking of antimicrobial efficacy was K > HH > VC > EC > Z. It is hypothesized that UV-C irradiation inactivates microorganisms by inducing thymine dimers and single-strand breaks in microbial DNA, while antioxidants may scavenge free radicals generated by UV exposure, inhibit microbial repair systems, and destabilize membrane proteins. Thus, the combined application of UV-C and antioxidants may exert a significant synergistic antimicrobial effect, which warrants further verification in future mechanistic studies [17,21]. These findings are consistent with previous reports. UV-C irradiation combined with Dean Vortex technology (which enhances fluid mixing and light distribution via swirling flow in coiled UV reactors) was effective in pineapple juice, as evidenced by a 5—log CFU/mL reduction of *S. typhimurium* at a dosage level of 13.8 mJ/cm^2^ [22]. UV-A combined with 0.1% fumaric acid lowered three pathogens in apple juice by ≥3.4 log [23]. Additionally, 14 mJ cm^−2^ UV-C + 300 mg kg^−1^ ascorbic acid delivered a 5-log reduction of *E. coli* in apple juice [12]. Together, these findings indicate that UV-C combined with antioxidants significantly extends the shelf-life of fruit juices. Similarly, Park et al. [24] demonstrated that thermo-sonication combined with 1% ascorbic acid achieved effective microbial inactivation (<0.5 log CFU/mL) in persimmon juice throughout 21 days of storage while maintaining color and viscosity.

#### 3.1.2. The Chroma Value

**Figure 2:**
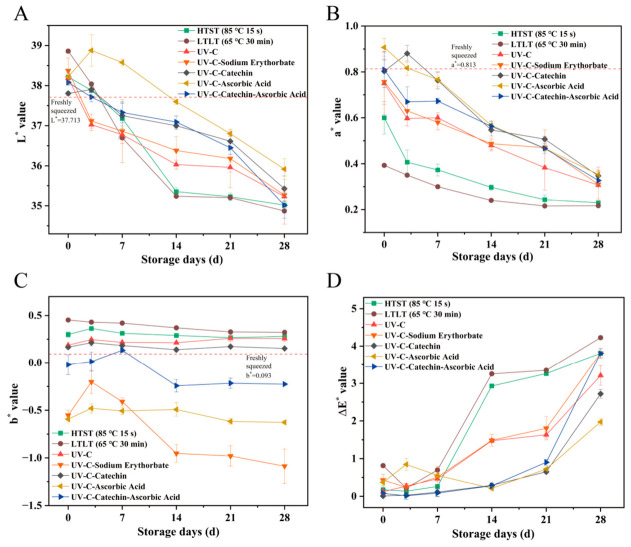
Color index changes of SSJ samples under different treatments during storage: (**A**) L* Value, (**B**) a* Value, (**C**) b* Value, (**D**) ΔE Value.

The colorimetric parameters of heat-treated, UV-C-treated, and freshly squeezed SSJ are shown in Figure 2. As shown in Figure 2A, Group LTLT exhibited the highest initial lightness (L*) among SSJ samples, followed by Group K, while the Group HH most closely resembled the values of freshly squeezed SSJ. From day 7 onward, all treatments lost brightness, but the pasteurized groups faded twice as fast as the UV-C groups (*p* < 0.05). The L* values became relatively stable from day 14 to day 28 [16].

Regarding the red–green coordinates (a*), positive values indicate the presence of red components, while negative values indicate green components. Figure 2B reveals that all treated samples contained only red components, with Groups VC and HH exhibiting more pronounced red components. Groups LTLT and HTST experienced accelerated degradation and isomerization of lycopene due to prolonged heating, causing their initial a* value to deviate from the maximum of freshly squeezed (0.813); this state was maintained throughout subsequent storage. However, by day 7 of storage, Group HH showed an increase in a* value, while all other groups exhibited decreases (*p* < 0.05). The characteristic color of SSJ is mainly attributed to carotenoids such as lycopene and carotene, while phenolic compounds including anthocyanins are also present as minor color-contributing components. The reduction in redness was associated with the oxidation of phenolic compounds. Group HH exhibited a significant synergistic antioxidant effect, showing a trend of initial increase followed by subsequent decrease.

The b* value corresponds to the yellow–blue axis. Figure 2C showed that the initial b* values of the K and VC differed significantly from those of freshly extracted SSJ. The Groups LTLT and HTST exhibited higher yellow component content, which remained largely unchanged throughout storage due to the thermal processing temperatures. In contrast, the Group EC consistently maintained values close to those of the freshly extracted sample. This phenomenon may stem from interactions between catechins and SSJ components, thereby enhancing the stability of carotenoids [25].

Finally, the total color difference (∆E) data in Figure 2D revealed that all treatment groups exhibited significant color changes from day 7 onward, with an overall upward trend. It has been reported that polyphenol oxidase (PPO) exhibits poor stability at temperatures above 40 °C, with complete inactivation achieved through 10 min treatment at 70 °C. Notably, the initial color difference of the Group HTST was lower than that of Group LTLT. Although the inactivation of PPO during heating may contribute to color retention, the observed color difference between heated and unheated samples was relatively small (below 1 unit) and imperceptible to the human eye. In addition, the color change of heated samples increased significantly after day 14, indicating that besides PPO inactivation, microbial proliferation may also affect the color of SSJ during storage [26]. However, ΔE values for both Groups HTST and LTLT rose rapidly during the later storage phase, indicating more pronounced color deterioration. During storage, the ΔE values for Groups HH and EC remained lower than those for Group Z. This indicates that when juice undergoes UV-C light treatment, the oxidation of multiple phenolic substrates may generate brown pigments through persistent polymerization processes. The formation of brown pigments usually leads to an increase in a* values and particularly in b* values. Combining antioxidant treatment with UV-C light may better preserve the initial color of SSJ.

#### 3.1.3. Effects of Decontamination Methods on Key Quality Changes of SSJ During Storage

**Figure 3:**
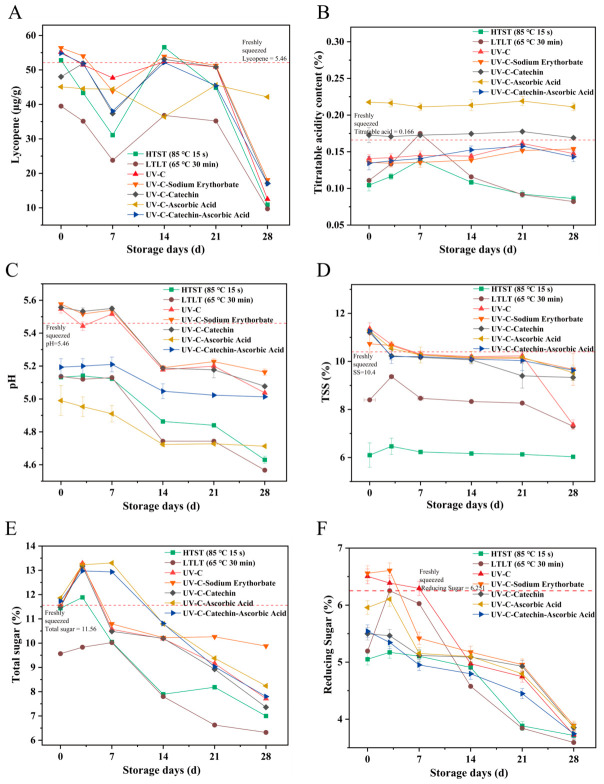
Quality changes of SSJ samples under different treatments during storage: (**A**) Lycopene content, (**B**) titratable acid content, (**C**) pH value, (**D**) TSS content, (**E**) total sugar content, (**F**) reducing sugar content.

Our data showed that the indicators of samples treated with different processing groups had distinct variation trends. The SSJ index of Group HTST was significantly lower than that of the freshly squeezed SSJ, indicating the greatest deterioration in quality, followed by Group LTLT (Figure 3D,F), while lycopene, pH, reducing sugar and soluble solids content increased after UV-C treatment (Figure 3A,C,D,F). Among them, the Group LTLT exhibited the most severe degradation of lycopene. This may be because the conjugated double bond structure of lycopene is disrupted by heat treatment, accelerating its oxidative degradation. UV-C treatment, however, may activate the photosensitive pigment synthesis pathway in SSJ, enhancing lycopene accumulation [20]. Additionally, antioxidants delay the oxidative degradation process by scavenging free radicals [21]. This aligns with existing research findings that, following nine weeks of cold storage, samples treated with ultraviolet C radiation exhibited higher levels of ascorbic acid, total phenolic compounds, and total antioxidant activity compared to heat-treated samples in pineapple and mango juice mixtures [27].

During storage, all samples showed significant fluctuations in lycopene, total sugar, and reducing sugar content over the four-week period (Figure 3A,E,F), with overall levels declining as storage time extended. Titratable acidity and soluble solids (Figure 3B,D) remained relatively stable with slight decreases. Among these, Groups HTST and LTLT values showed the most rapid decline (Figure 3A). When the storage time was extended to 28 days, lycopene levels decreased in all samples due to the cumulative effect of oxidative stress and accelerated lycopene degradation by microbial activity [28]. Figure 3B showed that the titratable acidity of freshly extracted SSJ from Group EC was most similar. Figure 3C demonstrated that Group VC exhibited the lowest pH variation and the most stable changes. This is mainly because ascorbic acid is a weak organic acid, which directly reduces the pH of the juice system and maintains high stability in the early stage of storage. With the extension of storage time, ascorbic acid gradually degrades, resulting in a slight increase and stabilization of pH. Group LTLT exhibited the most pronounced decline, which is most likely attributable to inadequate sterilization. Residual microorganisms proliferated during storage, leading to organic acid production and a consequent decrease in pH [10].

Figure 3D demonstrated no significant difference in total soluble solid (TSS) content among Groups Z, K, VC, EC, and HH (*p* > 0.05). Notably, Group Z showed a significant decrease in TSS content at storage end, while antioxidant groups maintained more stable levels [11]. The result mirrors the blueberry study of Ning et al. [3] where antioxidant addition under high pressure effectively inhibits component degradation [3]. Furthermore, previous studies have demonstrated that UV-C irradiation combined with mild heat treatment yields superior sensory quality in carrot–orange juice compared to pasteurized versions [29], and the combination of UV-C treatment with trans-cinnamaldehyde enhances the shelf-life of grapefruit juice without inducing any physicochemical or microbiological alterations, consistent with the findings of this study [30].

#### 3.1.4. Antioxidant Activity

**Figure 4:**
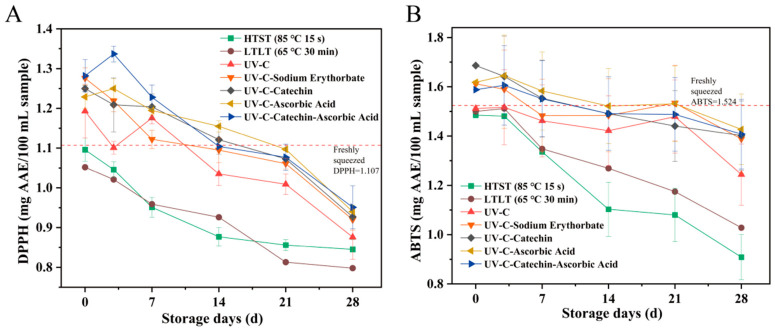
Antioxidant activity changes of SSJ under different treatments during 28-day storage: (**A**) DPPH radical scavenging activity, (**B**) ABTS radical scavenging activity.

Our data demonstrated that compared with Groups LTLT and HTST, samples treated with Z, K, VC, EC, and HH showed significantly enhanced antioxidant capacity as measured by DPPH and ABTS assays, with the Group HH showing the most notable improvement (Figure 4A,B). This is because UV-C is a non-thermal processing technology that causes less degradation of heat-sensitive antioxidants such as ascorbic acid, total phenols, and flavonoids. In contrast, thermal treatment readily destroys these bioactive compounds and inactivates antioxidant-related enzymes, which promotes the accumulation of phenolic compounds and enhances antioxidant capacity. These factors together lead to better preservation of antioxidant activity in UV-C treated samples than in thermally processed samples [31]. The UV-C plus antioxidant groups were superior to Group Z, and Group HH exhibited the optimal antioxidant activity, which can be explained by the fact that UV-C interacts with catechin and ascorbic acid. The antioxidant properties of catechin enable direct free radical scavenging [32]. This interaction creates a synergistic effect: ascorbic acid enhances catechin regeneration, thereby maintaining its antioxidant efficacy. Compared to Groups LTLT and HTST, this combined approach significantly boosts DPPH and ABTS values [3]. Like the trend of lycopene in Figure 3A, it indicates that the antioxidant activity in fresh juice mainly stems from its polyphenol content. The ABTS and DPPH tests showed no significant difference between UV-C combined with different antioxidants [33].

### 3.2. Effect of Different Treatments on Volatile Changes of SSJ During Storage

#### 3.2.1. GC–MS Analysis

GC-MS resolved 62 aroma compounds: 17 ketones, seven furans, eight aldehydes, 11 esters, four alcohols, four benzenoids and 11 miscellaneous volatiles [19,34]. The Group HTST detected 9 key substances, Group Z detected 13, Group HH detected 12, and Group K detected as many as 24 (Table S1). This data indicates that UV-C, especially when combined with antioxidants, can preserve a broader volatile spectrum than thermal pasteurization.

#### 3.2.2. Multivariate Statistical Analysis

**Figure 5:**
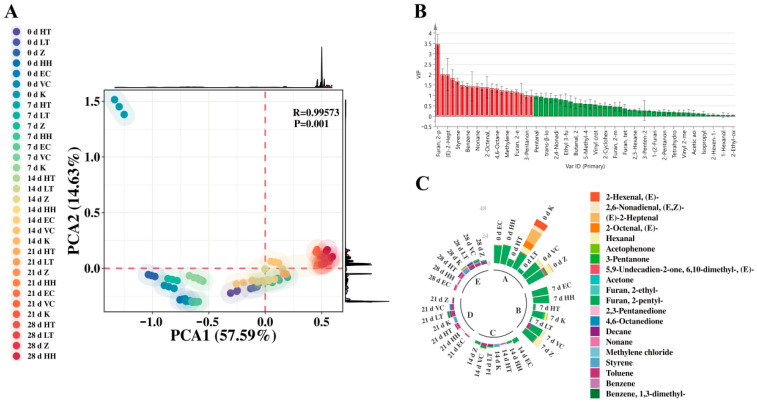
Multivariate statistical analysis of volatile flavor compounds in SSJ under different treatments during storage: (**A**) PCA analysis, (**B**) VIP plot, (**C**) distribution of volatile flavor compounds in VIP > 1 circular diagram. A is plotted according to the contents of volatile compounds in all samples, where the different colored points represent different sterilization methods. The horizontal coordinates in B represent volatile compounds, and the vertical coordinates represent VIP values, with the red part of the compounds for VIP > 1 and the green part of the compounds for VIP < 1. C clearly presents the proportion and distribution characteristics of various substances.

Peripheral PCA analysis was conducted on volatile flavor compounds in SSJ across different treatment groups (Figure 5A). PCA1 accounted for 57.59% of the variance, with model parameters R = 0.99573 and *p* = 0.001, indicating high model fidelity and reliable results. Results indicated that the Groups HTST and LTLT clustered closely in the PCA diagram, exhibiting significant positive shifts along the PCA1 axis with prolonged storage duration [10,20], whereas Group Z showed minimal flavor variation and distribution of the antioxidant group, and Group Z indicated that UV-C exposure had minimal impact on flavor compounds [35]. Concurrently, samples from the same treatment group and storage duration clustered well. The low relative deviation across the three replicates indicated excellent experimental reproducibility, further validating the reliability of the PCA results.

Figure 5B VIP analysis revealed key volatile compounds influencing flavor changes during SSJ storage under different treatments. Results indicated 19 compounds with VIP values exceeding 1 [36]. These comprised: one alcohol, six ketones, five aldehydes, one furan, three aromatic hydrocarbons, two alkanes, and one halogenated hydrocarbon. Previous studies have confirmed that 2-pentylfuran is the signature compound responsible for the characteristic flavor, originating from the enzymatic oxidation of linoleic acid. Its concentration directly determines the fresh flavor profile of watermelon juice [20] (Chantakun et al. 2022). Hexanal and (E)-2-heptenal, both belonging to the C6/C7 aldehyde group, are primary contributors to the grassy and fruity aromas in watermelon juice. Research indicates these aldehydes are highly heat-sensitive, with significant degradation occurring at temperatures above 60 °C. Aromatic hydrocarbons such as toluene (VIP = 1.820) and styrene (VIP = 1.689), though not characteristic flavor compounds of watermelon, have been identified in studies as indicators of flavor deterioration in heat-processed foods [37]. These primarily originate from the thermal degradation of carbohydrates and Maillard reactions. Furthermore, 3-pentanone holds particular significance in distinguishing processing methods, as ketones primarily originate from the β-oxidation of fatty acids, and their increased levels typically accompany flavor deterioration [38].

The VIP distribution map in Figure 5C visually illustrates differences in volatile compounds. The characteristic flavor compound 2-pentylfuran was present in all treatment groups at day 0, with the highest concentration observed in the Group HH. As storage time increased, the corresponding sector proportion of this compound significantly decreased over time in both the HTST and LTLT groups, whereas in Group Z (e.g., 21 d Z), the decrease in this compound’s proportion was smaller. High-temperature processing accelerates the degradation of flavor compounds; this aligns with existing research indicating [35,39]. Furthermore, during storage, the deterioration indicators toluene and styrene showed significantly smaller increases in Group Z and the antioxidant groups compared to Groups HTST and LTLT [10,21]. In summary, the combination of UV-C and antioxidants demonstrates excellent flavor-preserving efficacy, with Group HH exhibiting the most favorable results.

#### 3.2.3. Cluster Heatmap Analysis of Volatile Flavor Compounds During Storage Periods of SSJ Subjected to Different Treatments

**Figure 6:**
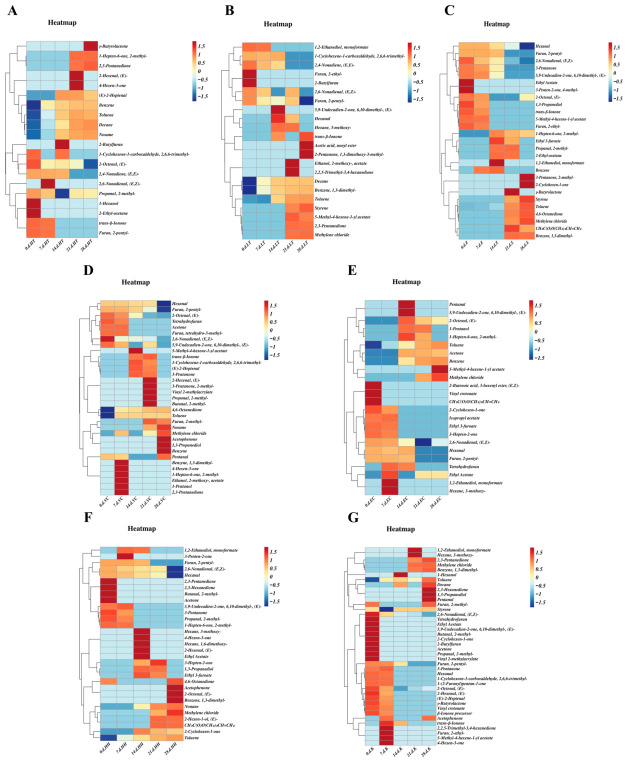
Analysis of volatile organic compounds. Heatmaps of volatile flavor compounds in SSJ under different treatments during storage: (**A**) Group HTST heatmap, (**B**) Group LTLT heatmap, (**C**) Group Z heatmap, (**D**) Group VC heatmap, (**E**) Group EC heatmap, (**F**) Group HH heatmap, (**G**) Group K heatmap. The redder color in the heatmap represents the relatively higher compound content, and the bluer color represents the relatively lower compound content.

Cluster heatmaps displayed that the high-temperature group and the non-high-temperature group differed significantly (Figure 6A,B). Groups Z, K, VC, EC and HH color blocks exhibited deep hues with minimal variation (Figure 6C–G). Results indicated that Group HTST exhibited the lowest initial concentration of key flavor compounds in SSJ; the same phenomenon was observed in orange juice [6]. This indicates that high-temperature processing accelerates the degradation of furan compounds, leading to the formation of aromatic hydrocarbon undesirable compounds [20]. Therefore, HTST and LTLT processes significantly influence the original flavor characteristics of SSJ [40].

As shown in Figure 6D–G, the group combining UV-C irradiation with antioxidants exhibited better stability than Group Z. This indicates that the combined use of UV irradiation and antioxidants significantly enhances stability. This demonstrated that catechin and ascorbic acid inhibit the oxidative degradation of characteristic flavor compounds by scavenging free radicals, particularly reflecting the synergistic antioxidant effects of the composite antioxidants [41].

#### 3.2.4. OPLS-DA Analysis Based on Flavor Changes in Different Samples During Storage

**Figure 7:**
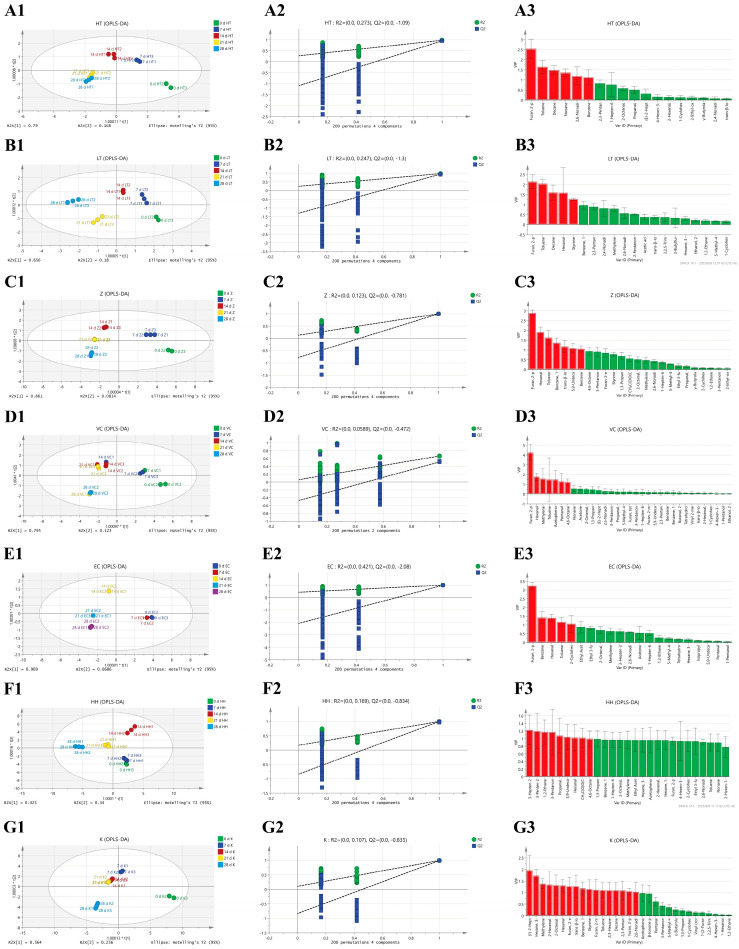
(1) OPLS-DA, (2) model validation and (3) VIP plot of compounds in SSJ under different treatments during storage: (**A**) Group HTST, (**B**) Group LTLT, (**C**) Group Z, (**D**) Group VC, (**E**) Group EC, (**F**) Group HH, (**G**) Group K. Permutation test of SSJ OPLS-DA up to 200 times for different sterilization methods.

To further elucidate flavor compound differences between processed samples, this study employed OPLS-DA modeling to analyze comparisons between thermally processed and non-thermally processed SSJ (Figure 7A1–G1). The reliability of the model was verified by a combination of the OPLS-DA score plots (Figure 7A1–G1) and permutation test plots (Figure 7A2–G2). The permutation test was considered valid when both regression lines had positive slopes and the intercept of Q2 was ≤0.05. The results showed that all permutation tests in Figure 7A2–G2 exhibited positive slopes; however, the intercept of Q2 in some groups slightly exceeded 0.05, suggesting mild overfitting. Thus, caution should be exercised regarding potential false-positive results.

Taking Group EC (Figure 7E1,E2) as an example, in the OPLS-DA score plot, the first principal component (t[1]) explained 90.9% of the between-group variation, while the second component (t[2]) explained 6.86%. The cumulative explanation rate of the two components reached 97.76%, indicating that the model had an extremely strong capacity to capture the overall differences among samples. The corresponding permutation test (Figure 7E2) showed a positive slope for the regression line and an intercept of Q2 < 0, which met the criteria for a valid permutation test. This confirmed that the model was not severely overfitted and possessed satisfactory interpretability and predictability. Through the score plot analysis of the OPLS-DA model in Figure 7A1–G1, combined with a comprehensive assessment of the importance of projected variables (VIP ≥ 1) in Figure 7A3–G3, furan, 2-pentyl-, and hexanal were identified as a significant differential compound for SSJ; whereas toluene and decane were regarded as key markers distinguishing heat-treated samples (HTST, LTLT) from non-heat-treated samples (Z, K, VC, EC, HH).

Groups HTST and LTLT exhibited a significantly greater displacement along the principal component axis (Figure 7A1,B1) during storage time compared to the other groups. Group HH exhibited a high degree of aggregation (Figure 7F1), primarily attributable to the reduced thermal loss of heat-sensitive compounds (such as 2-pentylfuran and hexanal) during storage due to the absence of heat treatment, coupled with the synergistic action of antioxidants effectively controlling the accumulation of degraded compounds. This conclusion is fully consistent with the results of PCA [18]. Groups HH and K contained a higher number of unique compounds with VIP values exceeding 1 (Figure 7F3,G3). Results indicated that the Group HH yielded eight compounds with VIP > 1, predominantly ketones (four compounds), followed by aldehydes (two compounds: 2-methylpropanal, hexanal), esters (one, 5-hexenoic acid 1-ethyl ester), and alcohol derivatives (one, ethylene glycol monomethyl ether). These categories predominantly correspond to natural flavoring components in watermelon juice, enhancing the juice’s body and layered fruit aromas [37]. Group K yielded 17 VIP > 1 compounds. The substance categories are complex and include oxidation by-products and potential risk substances. In contrast, Group K exhibited excessive accumulation of trans-2-heptenal, generating a pronounced ‘green grassy flavor’ that showed significant negative correlation with the ‘sweet aroma’ of watermelon juice. The presence of compounds such as dichloromethane also introduced potential irritation and ‘chemical taste’ risks, resulting in lower consumer acceptance compared to Group HH. Group K exhibited pronounced flavor defects and stability risks, which could potentially be mitigated by adding catechin and ascorbic acid.

## 4. Conclusions

This study compared the impact of UV-C non-thermal treatment and pasteurization on the quality and flavor compounds of fresh SSJ by testing multiple physicochemical indicators and detecting flavor changes during storage. Results indicate that key quality indicators such as lycopene content were higher in UV-C-treated samples than in freshly squeezed SSJ. For microbial stability, Group K (UV-C + sodium erythorbate) achieved complete bacteriostasis; no colonies were recovered at any sampling point. LTLT, by contrast, failed after day 3. The trial was purposely run under high-challenge conditions. Under normal industrial hygiene, the shelf-life advantage of the UV-C hurdles would be even greater. Immediately after treatment, the UV-C groups exhibited 15–25% higher lycopene, total phenolics, and DPPH/ABTS antioxidant capacity compared to fresh juice, likely due to UV-induced cell wall disruption enhancing extractability. In contrast, both thermal treatments caused immediate losses of 20–30%. For chemical quality, during storage the UV-C juices lost quality attributes more slowly; HH retained 92% of its initial antioxidant activity after 4 weeks. For aroma integrity, volatile compounds were analyzed using GC-MS, identifying 62 key active aromatic components. Multivariate statistics (PCA, OPLS-DA) showed that 2-pentylfuran and hexanal were chemical signatures of fresh SSJ, whereas toluene and decane were reliable markers of thermal impact. UV-C juices started with higher concentrations of the positive odorants and developed fewer off-notes. HH displayed the smallest overall drift, displaying superior flavor stability. Overall, UV-C combined with natural antioxidants, especially the catechin-ascorbic acid blend, delivers a safe, clean-label, non-thermal process that outperforms pasteurization in every critical quality dimension. Future studies should optimize the dose antioxidant ratio and validate this strategy under industrial continuous flow conditions to facilitate commercial adoption. In addition, systematic and comprehensive sensory evaluations encompassing appearance, aroma, taste, and overall acceptability will be conducted in follow-up studies to further assess the practical applicability of this treatment.

## Data Availability

The original contributions presented in this study are included in the article/Supplementary Materials. Further inquiries can be directed to the corresponding author.

## Supplementary Materials

The following supporting information can be downloaded at https://www.mdpi.com/article/10.3390/foods15091485/s1, Table S1. Qualitative and quantitative results of 62 aromatic compounds determined by GC-MS.
