# Supplementary material for: Effects of UV-C Combined with Different Antioxidants on Storage Quality and Flavor of Selenium-Sand Melon Juice
Source: Foods. 2026 Apr 24;15(9):1485. doi: 10.3390/foods15091485 (PMC13163787; doi:10.3390/foods15091485)
Supplement: Supplementary file 1 [file foods-15-01485-s001.zip › foods-4237745-supplementary.pdf]

Supplementary Material

Table S1. Qualitative and quantitative results of 62 aromatic compounds determined by GC-MS

**Compound name**	**Volatile compound content (μg/mL)**																																		
	0dHT	0dLT	0dZ	0dHH	0dEC	0dVC	0dK	7dHT	7dLT	7dZ	7dHH	7dEC	7dVC	7dK	14dHT	14dLT	14dZ	14dHH	14dEC	14dVC	14dK	21dHT	21dLT	21dZ	21dHH	21dEC	21dVC	21dK	28dHT	28dLT	28dZ	28dHH	28dEC	28dVC	28dK
1-Hexanol	0.0115	0.0000	0.0000	0.0000	0.0000	0.0000	0.0000	0.0000	0.0000	0.0000	0.0000	0.0000	0.0000	0.0000	0.0000	0.0000	0.0000	0.0000	0.0000	0.0000	0.0012	0.0000	0.0000	0.0000	0.0000	0.0000	0.0000	0.0000	0.0000	0.0000	0.0000	0.0000	0.0000	0.0000	0.0000
1-Pentanol	0.0000	0.0000	0.0000	0.0000	0.0000	0.0000	0.0000	0.0000	0.0000	0.0000	0.0000	0.0000	0.0014	0.0000	0.0000	0.0000	0.0000	0.0000	0.0004	0.0000	0.0000	0.0000	0.0000	0.0000	0.0000	0.0003	0.0000	0.0000	0.0000	0.0000	0.0000	0.0000	0.0000	0.0000	0.0000
5-Methyl-4-hexene-1-ylacetate	0.0000	0.0000	0.1452	0.0000	0.0000	0.0000	0.0000	0.0000	0.0000	0.0994	0.0000	0.0000	0.0000	0.1910	0.0000	0.0000	0.0000	0.0000	0.0000	0.0606	0.0000	0.0000	0.0093	0.0000	0.0000	0.0000	0.0000	0.0000	0.0000	0.0045	0.0000	0.0000	0.0043	0.0000	0.0000
1,3-Propanediol	0.00	0.00	0.03	0.00	0.00	0.00	0.00	0.00	0.00	0.00	0.00	0.00	0.00	0.00	0.00	0.00	0.00	0.00	0.00	0.00	0.00	0.00	0.00	0.00	0.00	0.00	0.00	0.00	0.00	0.00	0.00	0.00	0.00	0.00	0.00

	0	0	5	0	0	0	0	0	0	1	0	0	0	0	0	0	0	0	0	0	0	0	0	0	0	0	0	0	0	0	0	0	0	9	9
	0	0	9	0	0	0	0	0	0	7	0	0	0	0	0	0	0	1	0	0	0	0	0	0	1	0	0	0	0	0	0	0	0	8	8
	0	0	4	0	0	0	0	0	0	3	0	0	0	0	0	0	0	4	0	0	0	0	0	0	1	0	0	0	0	0	0	0	0	4	4
	0.	0.	0.	0.	0.	0.	0.	0.	0.	0.	0.	0.	0.	0.	0.	0.	0.	0.	0.	0.	0.	0.	0.	0.	0.	0.	0.	0.	0.	0.	0.	0.	0.	0.	0.
	0	0	0	0	0	0	0	0	0	0	0	0	0	0	0	0	0	0	0	0	0	0	0	0	0	0	0	0	0	0	0	0	0	0	0
2-Hexen-1-ol,	0	0	0	0	0	0	0	0	0	0	0	0	0	0	0	0	0	0	0	0	0	0	0	0	0	0	0	0	0	0	0	0	0	0	0
(E)-	0	0	0	0	0	0	0	0	0	0	0	0	0	0	0	0	0	0	0	0	0	0	0	0	4	0	0	0	0	0	0	3	0	0	0
	0	0	0	0	0	0	0	0	0	0	0	0	0	0	0	0	0	0	0	0	0	0	0	0	2	0	0	0	0	0	0	6	0	0	0
	0.	0.	0.	0.	0.	0.	0.	0.	0.	0.	0.	0.	0.	0.	0.	0.	0.	0.	0.	0.	0.	0.	0.	0.	0.	0.	0.	0.	0.	0.	0.	0.	0.	0.	0.
	2	0	0	0	0	0	8	0	0	0	0	0	0	0	0	0	0	0	0	0	0	0	0	0	0	0	0	0	0	0	0	0	0	0	0
Propanal,2-m	1	0	0	5	0	0	7	5	0	0	1	0	0	0	0	0	1	0	0	0	0	1	0	1	0	0	7	0	0	0	0	0	0	0	0
ethyl-	4	0	0	7	0	0	2	1	0	0	4	0	0	0	0	0	0	0	0	0	0	2	0	1	0	0	9	0	8	0	0	0	0	0	0
	5	0	0	8	0	0	0	6	0	0	7	0	0	0	0	0	4	0	0	0	0	0	0	2	0	0	1	0	6	0	0	0	0	0	0
	0.	0.	0.	0.	0.	0.	5.	0.	0.	0.	0.	0.	0.	0.	0.	0.	0.	0.	0.	0.	0.	0.	0.	0.	0.	0.	0.	0.	0.	0.	0.	0.	0.	0.	0.
	0	0	0	0	0	0	8	0	0	0	0	0	0	0	0	0	0	0	0	0	0	0	0	0	0	0	0	0	0	0	0	0	0	0	0
2-Hexenal,(E	0	0	0	0	0	0	6	0	0	0	0	0	0	2	0	0	0	0	0	0	0	0	0	0	0	0	1	0	0	0	0	0	0	0	0
)-	0	0	0	0	0	0	1	0	0	0	0	0	0	5	0	0	0	1	0	0	0	1	0	0	0	0	2	0	0	0	0	0	0	0	0
	0	0	0	0	0	0	3	0	0	0	0	0	0	0	0	0	0	0	0	0	0	2	0	0	0	0	0	0	0	0	0	0	0	0	0
	0.	0.	0.	0.	0.	0.	3.	0.	0.	0.	0.	0.	0.	0.	0.	0.	0.	0.	0.	0.	0.	0.	0.	0.	0.	0.	0.	0.	0.	0.	0.	0.	0.	0.	0.
	0	1	2	2	4	1	4	1	0	1	1	3	0	1	0	0	0	1	0	0	0	0	0	0	0	0	0	0	0	0	0	0	0	0	0
2,6-Nonadien	0	0	5	0	2	1	5	9	1	0	4	5	1	0	0	0	5	1	0	1	1	0	2	1	9	0	0	1	0	0	0	0	0	0	0
al,(E,Z)-	0	0	6	2	0	2	4	8	2	3	1	7	1	2	0	0	5	4	7	0	4	0	4	1	6	0	8	1	0	0	5	6	1	3	3
	0	1	3	0	4	5	0	8	5	6	3	6	5	1	0	0	4	4	9	6	7	0	5	4	5	0	2	6	0	0	6	1	9	6	6
	0.	0.	0.	0.	0.	0.	1	0.	0.	0.	0.	0.	0.	0.	0.	0.	0.	0.	0.	0.	0.	0.	0.	0.	0.	0.	0.	0.	0.	0.	0.	0.	0.	0.	0.
(E)-2-Hepten	0	0	0	0	0	0	1.	0	0	0	0	0	0	1	0	0	0	0	0	1	0	0	0	0	0	0	0	0	0	0	0	0	0	0	0
al	0	0	0	0	0	0	7	0	0	0	0	0	0	1	2	0	0	0	0	0	0	1	0	0	0	0	3	0	0	0	0	0	0	0	0

	0	0	0	0	0	0	3	0	0	0	0	0	0	7	3	0	0	0	0	1	0	8	0	0	0	0	1	0	4	0	0	0	0	0	0
	0	0	0	0	0	0	0	0	0	0	0	0	0	5	4	0	0	0	0	1	0	5	0	0	0	0	7	0	6	0	0	0	0	0	0
							0																												
	0.	0.	0.	0.	0.	0.	0.	0.	0.	0.	0.	0.	0.	0.	0.	0.	0.	0.	0.	0.	0.	0.	0.	0.	0.	0.	0.	0.	0.	0.	0.	0.	0.	0.	0.
	0	0	0	0	0	0	0	0	0	0	0	0	0	0	0	0	0	0	0	0	0	0	0	0	0	0	0	0	0	0	0	0	0	7	7
Pentanal	0	0	0	0	0	2	0	0	0	0	0	0	1	0	0	0	0	0	0	0	0	0	0	0	0	0	0	0	0	0	0	0	0	4	4
	0	0	0	0	0	2	0	0	0	0	0	0	1	0	0	0	0	0	1	0	0	0	0	0	0	0	0	0	0	0	0	0	0	1	1
	0	0	0	0	0	1	0	0	0	0	0	0	7	0	0	0	0	0	3	0	0	0	0	0	0	0	0	0	0	0	0	0	0	3	3
	0.	0.	0.	0.	0.	0.	0.	0.	0.	0.	0.	0.	0.	0.	0.	0.	0.	0.	0.	0.	0.	0.	0.	0.	0.	0.	0.	0.	0.	0.	0.	0.	0.	0.	0.
1-Cyclohexene-1-carboxaldehyde,2,6,6-trimethyl-	0	0	0	0	0	0	0	0	0	0	0	0	0	0	0	0	0	0	0	0	0	0	0	0	0	0	0	0	0	0	0	0	0	0	0
	0	1	0	0	0	0	6	0	0	0	0	0	0	5	0	1	0	0	0	0	0	0	0	0	0	0	0	0	0	0	0	0	0	0	0
	2	2	0	0	0	0	4	0	9	0	0	0	0	2	2	2	0	0	0	3	0	0	0	0	0	0	1	0	0	0	0	0	0	0	0
	7	8	0	0	0	0	7	0	8	0	0	0	0	1	3	2	0	0	0	5	0	0	0	0	0	0	0	0	0	0	0	0	0	0	0
	0.	0.	0.	0.	0.	0.	5.	0.	0.	0.	0.	0.	0.	0.	0.	0.	0.	0.	0.	0.	0.	0.	0.	0.	0.	0.	0.	0.	0.	0.	0.	0.	0.	0.	0.
	1	0	4	0	0	2	5	0	0	1	0	0	1	4	0	0	0	0	1	0	0	0	0	0	0	0	0	0	0	0	0	0	0	0	0
2-Octenal,(E)-	2	0	2	0	0	4	2	0	0	4	0	0	6	2	0	0	0	0	2	5	0	0	0	0	0	0	1	0	0	0	0	0	0	1	1
	5	0	5	0	0	1	3	1	0	7	0	0	5	6	2	0	0	0	4	1	1	0	0	0	0	5	3	4	0	0	2	4	1	1	1
	6	0	8	0	0	0	0	5	0	1	0	0	9	6	4	0	0	0	0	9	1	9	0	0	0	4	4	8	0	0	3	6	2	2	2
	0.	0.	4.	0.	0.	3.	5.	0.	0.	4.	0.	0.	2.	1.	0.	0.	0.	0.	0.	1.	0.	0.	0.	0.	0.	0.	0.	0.	0.	0.	0.	0.	0.	0.	0.
	0	0	9	8	4	1	1	0	0	7	3	2	4	7	0	6	2	0	5	0	0	0	0	0	0	0	8	0	0	0	0	0	0	0	0
Hexanal	0	0	8	5	0	7	3	0	0	2	4	1	4	9	0	6	8	9	2	7	0	0	0	1	2	0	7	0	0	0	0	0	0	0	0
	0	0	5	6	5	5	8	0	0	3	5	4	1	6	0	0	9	8	4	2	0	0	4	0	4	0	7	0	0	0	0	0	0	0	0
	0	0	0	0	1	1	0	0	0	6	1	3	3	3	0	0	4	5	1	0	0	0	4	2	5	0	2	0	0	0	0	0	0	0	0
	0.	0.	0.	0.	0.	0.	0.	0.	0.	0.	0.	0.	0.	0.	0.	0.	0.	0.	0.	0.	0.	0.	0.	0.	0.	0.	0.	0.	0.	0.	0.	0.	0.	0.	0.
Butanal,2-methyl-	0	0	0	1	0	0	0	0	0	0	0	0	0	0	0	0	0	0	0	0	0	0	0	0	0	0	0	0	0	0	0	0	0	0	0
	0	0	0	2	0	0	4	0	0	0	0	0	0	0	0	0	0	0	0	0	0	0	0	0	0	0	2	0	0	0	0	0	0	0	0

	0	0	0	7	0	0	2	0	0	0	0	0	0	0	0	0	0	0	0	0	0	0	0	0	0	0	4	0	0	0	0	0	0	0	0
	0	0	0	8	0	0	6	0	0	0	0	0	0	0	0	0	0	0	0	0	0	0	0	0	0	0	5	0	0	0	0	0	0	0	0
	0.	0.	0.	0.	0.	0.	0.	0.	0.	0.	0.	0.	0.	0.	0.	0.	0.	0.	0.	0.	0.	0.	0.	0.	0.	0.	0.	0.	0.	0.	0.	0.	0.	0.	0.
Ethyl3-furoate	0	0	0	0	4	0	0	0	0	0	0	3	0	0	0	0	0	1	0	0	0	0	0	0	0	0	0	0	0	0	0	0	0	0	0
	0	0	0	0	9	0	0	0	0	0	0	1	0	0	0	0	1	1	0	0	0	0	0	0	2	0	0	0	0	0	0	0	0	0	0
	0	0	0	0	9	0	0	0	0	0	0	2	0	0	0	0	1	9	0	0	0	0	0	1	6	0	0	0	0	0	0	0	0	0	0
	0	0	0	0	5	0	0	0	0	0	0	4	0	0	0	0	6	8	0	0	0	0	0	0	4	0	0	0	0	0	0	0	0	0	0
	0.	0.	0.	0.	0.	0.	0.	0.	0.	0.	0.	0.	0.	0.	0.	0.	0.	0.	0.	0.	0.	0.	0.	0.	0.	0.	0.	0.	0.	0.	0.	0.	0.	0.	0.
Vinyl2-methylacrylate	0	0	0	0	0	0	0	0	0	0	0	0	0	0	0	0	0	0	0	0	0	0	0	0	0	0	0	0	0	0	0	0	0	0	0
	0	0	0	0	0	0	1	0	0	0	0	0	0	0	0	0	0	0	0	0	0	0	0	0	0	0	1	0	0	0	0	0	0	0	0
	0	0	0	0	0	0	1	0	0	0	0	0	0	0	0	0	0	0	0	0	0	0	0	0	0	0	3	0	0	0	0	0	0	0	0
	0	0	0	0	0	0	2	0	0	0	0	0	0	0	0	0	0	0	0	0	0	0	0	0	0	0	6	0	0	0	0	0	0	0	0
	0.	0.	0.	0.	0.	0.	0.	0.	0.	0.	0.	0.	0.	0.	0.	0.	0.	0.	0.	0.	0.	0.	0.	0.	0.	0.	0.	0.	0.	0.	0.	0.	0.	0.	0.
γ-Butyrolactone	0	0	0	0	0	0	2	0	0	0	0	0	0	0	0	0	0	0	0	0	0	0	0	0	0	0	0	0	0	0	0	0	0	0	0
	0	0	0	0	0	0	1	0	0	0	0	0	0	1	0	0	0	0	0	0	0	0	0	0	0	0	0	0	0	0	0	0	0	0	0
	0	0	0	0	0	0	5	0	0	0	0	0	0	7	0	0	0	0	0	0	0	0	0	1	0	0	0	0	1	0	0	0	0	0	0
	0	0	0	0	0	0	9	0	0	0	0	0	0	8	0	0	0	0	0	0	0	0	0	2	0	0	0	0	4	0	0	0	0	0	0
	0.	0.	0.	0.	0.	0.	0.	0.	0.	0.	0.	0.	0.	0.	0.	0.	0.	0.	0.	0.	0.	0.	0.	0.	0.	0.	0.	0.	0.	0.	0.	0.	0.	0.	0.
Aceticacid,nonylester	0	0	0	0	0	0	0	0	0	0	0	0	0	0	0	0	0	0	0	0	0	0	0	0	0	0	0	0	0	0	0	0	0	0	0
	0	0	0	0	0	0	0	0	0	0	0	0	0	0	0	0	0	0	0	0	0	0	0	0	0	0	0	0	0	3	0	0	0	0	0
	0	0	0	0	0	0	0	0	0	0	0	0	0	0	0	0	0	0	0	0	0	0	0	0	0	0	0	0	0	3	0	0	0	0	0
	0	0	0	0	0	0	0	0	0	0	0	0	0	0	0	0	0	0	0	0	0	0	0	0	0	0	0	0	0	5	0	0	0	0	0
	0.	0.	0.	0.	0.	0.	0.	0.	0.	0.	0.	0.	0.	0.	0.	0.	0.	0.	0.	0.	0.	0.	0.	0.	0.	0.	0.	0.	0.	0.	0.	0.	0.	0.	0.
Ethanol,2-methoxy-,acetate	0	0	0	0	0	0	0	0	0	0	0	0	0	0	0	0	0	0	0	0	0	0	0	0	0	0	0	0	0	0	0	0	0	0	0
	0	0	0	0	0	0	0	0	0	0	0	0	0	0	0	0	0	0	0	0	0	0	0	0	0	0	0	0	0	0	0	0	0	0	0
	0	0	0	0	0	0	0	0	0	0	0	0	1	0	0	0	0	0	0	0	0	0	8	0	0	0	0	0	0	0	0	0	0	0	0

	0	0	0	0	0	0	0	0	0	0	0	0	2	0	0	0	0	0	0	0	0	0	9	0	0	0	0	0	0	0	0	0	0	0	0
	0.	0.	0.	0.	0.	0.	0.	0.	0.	0.	0.	0.	0.	0.	0.	0.	0.	0.	0.	0.	0.	0.	0.	0.	0.	0.	0.	0.	0.	0.	0.	0.	0.	0.	0.
	0	0	0	0	2	0	0	0	0	0	0	0	0	0	0	0	0	0	0	0	0	0	0	0	0	0	0	0	0	0	0	0	0	0	0
Vinylcrotonate	0	0	0	0	3	0	6	0	0	0	0	0	0	1	0	0	0	0	0	0	0	0	0	0	0	0	0	0	0	0	0	0	0	0	0
	0	0	0	0	1	0	3	0	0	0	0	0	0	2	0	0	0	0	0	0	0	0	0	0	0	0	0	0	0	0	0	0	0	0	0
	0	0	0	0	8	0	4	0	0	0	0	0	0	1	0	0	0	0	0	0	0	0	0	0	0	0	0	0	0	0	0	0	0	0	0
	0.	0.	0.	0.	0.	0.	0.	0.	0.	0.	0.	0.	0.	0.	0.	0.	0.	0.	0.	0.	0.	0.	0.	0.	0.	0.	0.	0.	0.	0.	0.	0.	0.	0.	0.
	0	0	0	0	4	0	0	0	0	0	0	0	0	0	0	0	0	0	0	0	0	0	0	0	1	0	0	0	0	0	0	1	0	0	0
Hex-5-en-1-yl acetate	0	0	0	0	1	0	0	0	0	0	0	0	0	0	0	0	0	0	0	0	0	0	0	5	9	0	0	0	0	0	0	5	0	0	0
	0	0	0	0	4	0	0	0	0	0	0	0	0	0	0	0	0	0	0	0	0	0	0	3	5	0	0	0	0	0	3	3	0	0	0
	0	0	0	0	2	0	0	0	0	0	0	0	0	0	0	0	0	0	0	0	0	0	0	0	4	0	0	0	0	0	6	2	0	0	0
	0.	0.	1.	0.	0.	0.	0.	0.	0.	0.	0.	0.	0.	0.	0.	0.	0.	0.	0.	0.	0.	0.	0.	0.	0.	0.	0.	0.	0.	0.	0.	0.	0.	0.	0.
	0	0	1	0	0	0	0	0	0	0	0	1	0	0	0	0	0	0	0	0	0	0	0	0	0	0	0	0	0	0	0	0	0	0	0
EthylAcetate	0	0	5	0	0	0	1	0	0	0	0	1	0	0	0	0	0	1	0	0	0	0	0	0	0	0	0	0	0	0	0	0	0	0	0
	0	0	5	0	0	0	1	0	0	0	0	4	0	0	0	0	0	4	0	0	0	0	0	0	0	1	0	0	0	0	0	0	1	0	0
	0	0	4	0	0	0	3	0	0	0	0	5	0	0	0	0	0	5	0	0	0	0	0	0	0	2	0	0	0	0	0	0	1	0	0
	0.	0.	0.	0.	0.	0.	0.	0.	0.	0.	0.	0.	0.	0.	0.	0.	0.	0.	0.	0.	0.	0.	0.	0.	0.	0.	0.	0.	0.	0.	0.	0.	0.	0.	0.
1,2-Ethanediol,monoformate	0	0	0	0	0	0	0	0	0	0	0	0	0	0	0	0	0	0	0	0	0	0	0	0	0	0	0	0	0	0	0	0	0	0	0
	0	1	0	0	0	0	0	0	1	0	1	1	0	0	0	0	0	0	0	0	0	0	0	0	0	0	0	0	0	0	0	0	0	0	0
	0	5	0	0	0	0	0	0	3	0	1	1	0	0	0	0	1	7	0	0	0	0	0	0	0	0	0	1	0	0	0	0	0	0	0
	0	4	0	0	0	0	0	0	5	0	4	5	0	0	0	0	4	4	0	0	0	0	0	0	0	0	0	1	0	0	0	0	0	0	0
	0.	0.	0.	0.	0.	0.	0.	0.	0.	0.	0.	0.	0.	0.	0.	0.	0.	0.	0.	0.	0.	0.	0.	0.	0.	0.	0.	0.	0.	0.	0.	0.	0.	0.	0.
	0	0	0	0	0	0	0	0	0	0	0	0	0	0	0	0	0	0	0	0	0	0	0	0	0	0	0	0	0	0	0	0	0	0	0
Isopropylacetate	0	0	0	0	0	0	0	0	0	0	0	0	0	0	0	0	0	0	0	0	0	0	0	0	0	0	0	0	0	0	0	0	0	0	0
	0	0	0	0	7	0	0	0	0	0	0	9	0	0	0	0	0	0	0	0	0	0	0	0	0	0	0	0	0	0	0	0	0	0	0
	0	0	0	0	5	0	0	0	0	0	0	7	0	0	0	0	0	0	0	0	0	0	0	0	0	0	0	0	0	0	0	0	0	0	0

	0.	0.	0.	0.	0.	0.	0.	0.	0.	0.	0.	0.	0.	0.	0.	0.	0.	0.	0.	0.	0.	0.	0.	0.	0.	0.	0.	0.	0.	0.	0.	0.	0.	0.	0.
2-Butenoicaci	0	0	0	0	3	0	0	0	0	0	0	0	0	0	0	0	0	0	0	0	0	0	0	0	0	0	0	0	0	0	0	0	0	0	0
d,3-hexenyles	0	0	0	0	5	0	0	0	0	0	0	0	0	0	0	0	0	0	0	0	0	0	0	0	0	0	0	0	0	0	0	0	0	0	0
ter,(E,Z)-	0	0	0	0	8	0	0	0	0	0	0	0	0	0	0	0	0	0	0	0	0	0	0	0	0	0	0	0	0	0	0	0	0	0	0
	0	0	0	0	1	0	0	0	0	0	0	0	0	0	0	0	0	0	0	0	0	0	0	0	0	0	0	0	0	0	0	0	0	0	0
	0.	0.	0.	0.	0.	0.	0.	0.	0.	0.	0.	0.	0.	0.	0.	0.	0.	0.	0.	0.	0.	0.	0.	0.	0.	0.	0.	0.	0.	0.	0.	0.	0.	0.	0.
	0	0	0	0	0	0	0	0	0	0	0	0	0	0	0	0	0	0	0	0	0	0	0	0	0	0	0	0	0	0	0	0	0	0	0
3-Heptanone,	0	0	0	0	0	0	0	0	0	0	0	0	0	0	0	0	0	0	0	0	0	0	0	0	0	0	0	0	0	0	0	0	0	0	0
6-methyl-	0	0	0	0	0	0	0	0	0	0	0	0	0	0	0	0	0	0	0	0	0	0	0	0	0	0	0	0	0	0	0	0	0	0	0
	0	0	0	0	0	0	0	0	0	0	0	0	0	0	0	0	0	0	0	0	0	0	0	0	0	0	0	0	0	0	0	0	0	0	0
	0.	0.	0.	0.	0.	0.	0.	0.	0.	0.	0.	0.	0.	0.	0.	0.	0.	0.	0.	0.	0.	0.	0.	0.	0.	0.	0.	0.	0.	0.	0.	0.	0.	0.	0.
	0	0	5	0	0	0	0	0	0	0	0	0	0	0	0	0	0	0	0	0	0	0	0	0	0	0	0	0	0	0	0	0	0	0	0
3-Penten-2-o	0	0	4	0	0	0	0	0	0	0	0	0	0	0	0	0	0	0	0	0	0	0	0	0	0	0	0	0	0	0	0	0	0	0	0
ne,4-methyl-	0	0	8	0	0	0	0	0	0	0	0	0	0	0	0	0	0	0	0	0	0	0	0	0	0	0	0	0	0	0	0	0	0	0	0
	0	0	1	0	0	0	0	0	0	0	0	0	0	0	0	0	0	0	0	0	0	0	0	0	0	0	0	0	0	0	0	0	0	0	0
	0.	0.	0.	0.	0.	0.	0.	0.	0.	0.	0.	0.	0.	0.	0.	0.	0.	0.	0.	0.	0.	0.	0.	0.	0.	0.	0.	0.	0.	0.	0.	0.	0.	0.	0.
	0	0	0	0	0	0	0	0	0	0	0	0	0	0	0	0	0	0	0	0	0	0	0	0	0	0	0	0	0	0	0	0	0	0	0
4-Hexen-3-on	0	0	0	0	0	0	0	0	0	0	0	0	0	0	0	0	0	0	0	0	0	0	0	0	0	0	0	0	0	0	0	0	0	0	0
e	0	0	0	0	0	0	0	0	0	0	0	0	4	6	0	0	0	1	0	0	0	1	0	0	0	0	0	0	0	0	0	0	0	0	0
	0	0	0	0	0	0	0	0	0	0	0	0	7	5	0	0	0	3	0	0	0	4	0	0	0	0	0	0	0	0	0	0	0	0	0
	0.	0.	0.	0.	0.	0.	0.	0.	0.	0.	0.	0.	0.	0.	0.	0.	0.	0.	0.	0.	0.	0.	0.	0.	0.	0.	0.	0.	0.	0.	0.	0.	0.	0.	0.
2-Pentanone,	0	0	0	0	0	0	0	0	0	0	0	0	0	0	0	0	0	0	0	0	0	0	0	0	0	0	0	0	0	0	0	0	0	0	0
1,3-dimethox	0	0	0	0	0	0	0	0	0	0	0	0	0	0	0	0	0	0	0	0	0	0	0	0	0	0	0	0	0	6	0	0	0	0	0
y-3-methyl-	0	0	0	0	0	0	0	0	0	0	0	0	0	0	0	0	0	0	0	0	0	0	0	0	0	0	0	0	0	6	0	0	0	0	0
	0	0	0	0	0	0	0	0	0	0	0	0	0	0	0	0	0	0	0	0	0	0	0	0	0	0	0	0	0	5	0	0	0	0	0
3-Pentanone,	0.	0.	0.	0.	0.	0.	0.	0.	0.	0.	0.	0.	0.	0.	0.	0.	0.	0.	0.	0.	0.	0.	0.	0.	0.	0.	0.	0.	0.	0.	0.	0.	0.	0.	0.

2-methyl-	0	0	0	0	0	0	0	0	0	0	0	0	0	0	0	0	0	0	0	0	0	0	0	0	0	0	0	0	0	0	0	0	0	0	0
	0	0	0	0	0	0	0	0	0	0	0	0	0	0	0	0	0	0	0	0	0	0	0	0	0	0	6	0	0	0	0	0	0	0	0
	0	0	0	0	0	0	0	0	0	0	0	0	0	0	0	0	0	0	0	0	0	0	0	0	0	0	7	0	0	0	1	0	0	0	0
	0	0	0	0	0	0	0	0	0	0	0	0	0	0	0	0	0	0	0	0	0	0	0	0	0	0	8	0	0	0	5	0	0	0	0
	0.	0.	0.	0.	0.	0.	0.	0.	0.	0.	0.	0.	0.	0.	0.	0.	0.	0.	0.	0.	0.	0.	0.	0.	0.	0.	0.	0.	0.	0.	0.	0.	0.	0.	0.
	0	0	0	2	0	0	0	0	0	0	1	0	0	0	0	0	0	0	0	0	0	1	0	0	0	0	0	0	0	0	0	0	0	0	0
1-Hepten-6-o	0	0	0	6	0	0	0	0	0	0	4	0	9	0	0	0	5	0	7	0	0	1	0	1	0	0	0	0	9	0	0	0	0	0	0
ne,2-methyl-	0	0	0	6	0	0	0	0	0	0	7	0	5	0	0	0	4	0	5	0	0	2	0	4	0	4	0	0	5	0	5	0	0	0	0
	0	0	0	8	0	0	0	0	0	0	1	0	1	0	0	0	1	0	6	0	0	1	0	3	0	5	0	0	6	0	2	0	0	0	0
	0.	0.	0.	0.	0.	0.	0.	0.	0.	0.	0.	0.	0.	0.	0.	0.	0.	0.	0.	0.	0.	0.	0.	0.	0.	0.	0.	0.	0.	0.	0.	0.	0.	0.	0.
	0	0	0	0	0	0	0	0	0	0	0	0	0	0	0	0	0	0	0	0	0	0	0	0	0	0	0	0	0	0	0	0	0	0	0
1-(2-Furanyl)	0	0	0	0	0	0	3	0	0	0	0	0	0	2	0	0	0	0	0	0	0	0	0	0	0	0	0	0	0	0	0	0	0	0	0
pentan-1-one	0	0	0	0	0	0	5	0	0	0	0	0	0	8	0	0	0	0	0	0	0	0	0	0	0	0	0	0	0	0	0	0	0	0	0
	0	0	0	0	0	0	7	0	0	0	0	0	0	5	0	0	0	0	0	0	0	0	0	0	0	0	0	0	0	0	0	0	0	0	0
	0.	0.	0.	0.	0.	0.	0.	0.	0.	0.	0.	0.	0.	1.	0.	0.	0.	0.	0.	0.	0.	0.	0.	0.	0.	0.	0.	0.	0.	0.	0.	0.	0.	0.	0.
	0	0	0	0	0	0	0	0	0	0	0	0	0	0	0	0	0	0	0	0	0	0	0	0	0	0	0	0	0	0	0	2	0	9	9
Acetophenon	0	0	0	0	0	0	0	0	0	0	0	0	0	7	0	0	0	0	0	0	0	0	0	0	0	0	0	0	0	0	0	1	0	8	8
e	0	0	0	0	0	0	0	0	0	0	0	0	0	8	0	0	0	0	0	0	0	0	0	0	0	0	0	0	0	0	0	5	0	5	5
	0	0	0	0	0	0	0	0	0	0	0	0	0	1	0	0	0	0	0	0	0	0	0	0	0	0	0	0	0	0	0	6	0	1	1
	0.	0.	0.	0.	0.	0.	0.	0.	0.	0.	0.	0.	0.	0.	0.	0.	0.	0.	0.	0.	0.	0.	0.	0.	0.	0.	0.	0.	0.	0.	0.	0.	0.	0.	0.
	0	0	0	0	2	0	0	0	0	0	0	1	0	0	0	0	0	0	0	0	0	0	0	0	0	0	0	0	0	0	0	0	0	0	0
3-Hepten-2-o	0	0	0	0	1	0	0	0	0	0	0	1	0	0	0	0	0	0	0	0	0	0	0	1	0	0	0	0	0	0	0	0	0	0	0
ne	0	0	0	0	9	0	0	0	0	0	0	0	0	0	0	0	0	1	0	0	0	0	0	6	0	0	0	0	0	0	0	0	0	0	0
	0	0	0	0	0	0	0	0	0	0	0	9	0	0	0	0	0	0	0	0	0	0	0	0	0	0	0	0	0	0	0	0	0	0	0
3-Pentanone	0.	0.	0.	0.	0.	0.	0.	0.	0.	0.	0.	0.	0.	0.	0.	0.	0.	0.	0.	0.	0.	0.	0.	0.	0.	0.	0.	0.	0.	0.	0.	0.	0.	0.	0.
	0	0	9	4	0	0	1	0	0	2	0	0	0	2	0	0	0	0	0	0	0	0	0	0	0	0	0	0	0	0	0	0	0	0	0

	0	0	6	5	0	0	2	0	0	2	2	0	0	7	0	0	0	0	0	4	0	0	0	0	0	0	2	0	0	0	0	0	0	0	0
	0	0	5	5	0	0	3	0	0	6	5	0	0	7	0	0	3	0	0	2	0	0	0	0	0	0	0	0	0	0	0	0	0	0	0
	0	0	4	1	0	0	8	0	0	0	8	0	0	4	0	0	6	0	0	2	0	0	0	0	0	0	2	0	0	0	0	0	0	0	0
	0.	0.	0.	0.	0.	0.	0.	0.	0.	0.	0.	0.	0.	0.	0.	0.	0.	0.	0.	0.	0.	0.	0.	0.	0.	0.	0.	0.	0.	0.	0.	0.	0.	0.	0.
	0	0	0	0	2	0	1	0	0	0	0	0	0	0	0	0	0	0	0	0	0	0	0	0	0	0	0	0	0	0	0	0	0	0	0
2-Cyclohexen-1-one	0	0	0	0	1	0	0	0	0	0	0	2	0	0	0	0	0	5	0	0	0	0	0	0	1	0	0	0	0	0	0	0	0	0	0
	0	0	0	0	5	0	2	0	0	0	0	1	0	0	0	0	0	6	0	0	0	0	0	0	1	0	0	0	0	0	2	5	0	0	0
	0	0	0	0	9	0	3	0	0	0	0	5	0	0	0	0	0	8	0	0	0	0	0	0	7	0	0	0	0	0	5	9	0	0	0
	0.	0.	0.	0.	0.	0.	0.	0.	0.	0.	0.	0.	0.	0.	0.	0.	0.	0.	0.	0.	0.	0.	0.	0.	0.	0.	0.	0.	0.	0.	0.	0.	0.	0.	0.
2,2,5-Trimeth	0	0	0	0	0	0	0	0	0	0	0	0	0	0	0	0	0	0	0	0	0	0	0	0	0	0	0	0	0	0	0	0	0	0	0
yl-3,4-hexane	0	0	0	0	0	0	0	0	0	0	0	0	0	1	0	0	0	0	0	0	0	0	2	0	0	0	0	0	0	0	0	0	0	0	0
dione	0	0	0	0	0	0	0	0	0	0	0	0	0	5	0	0	0	0	0	0	0	0	3	0	0	0	0	0	0	0	0	0	0	0	0
	0	0	0	0	0	0	0	0	0	0	0	0	0	6	0	0	0	0	0	0	0	0	3	0	0	0	0	0	0	0	0	0	0	0	0
	0.	0.	1.	0.	0.	0.	0.	0.	0.	0.	0.	0.	0.	0.	0.	0.	0.	0.	0.	0.	0.	0.	0.	0.	0.	0.	0.	0.	0.	0.	0.	0.	0.	0.	0.
	0	0	3	0	0	0	0	0	0	1	0	0	0	1	0	0	0	0	0	0	0	0	0	0	0	0	0	0	0	0	0	0	0	0	0
trans-β-Ionone	0	0	2	0	0	0	0	0	0	4	0	0	0	1	0	0	0	0	0	0	0	0	0	0	0	0	0	0	0	3	0	0	0	0	0
	2	0	5	0	0	0	0	1	0	4	0	0	0	1	0	5	0	0	0	3	1	0	0	0	0	0	5	0	0	3	0	0	0	0	0
	4	0	0	0	0	0	0	9	0	0	0	0	0	0	0	2	0	0	0	0	9	0	0	0	0	0	9	0	0	5	0	0	0	0	0
	0.	0.	0.	0.	0.	0.	0.	0.	0.	0.	0.	0.	0.	0.	0.	0.	0.	0.	0.	0.	0.	0.	0.	0.	0.	0.	0.	0.	0.	0.	0.	0.	0.	0.	0.
	0	0	0	0	0	0	1	0	0	0	0	0	0	0	0	0	0	0	0	0	0	0	0	0	0	0	0	0	0	0	0	0	0	0	0
β-Iononeprecursor	0	0	0	0	0	0	1	0	0	0	0	0	0	1	0	0	0	0	0	0	0	0	0	0	0	0	0	0	0	0	0	0	0	0	0
	0	0	0	0	0	0	2	0	0	0	0	0	0	4	0	0	0	0	0	0	0	0	0	0	0	0	0	0	0	0	0	0	0	0	0
	0	0	0	0	0	0	4	0	0	0	0	0	0	0	0	0	0	0	0	0	0	0	0	0	0	0	0	0	0	0	0	0	0	0	0
5,9-Undecadi	0.	0.	1.	0.	0.	0.	0.	0.	0.	0.	0.	0.	0.	0.	0.	0.	0.	0.	0.	0.	0.	0.	0.	0.	0.	0.	0.	0.	0.	0.	0.	0.	0.	0.	0.
en-2-one,6,10	0	0	6	0	0	0	6	0	0	5	0	0	0	0	0	0	0	0	0	0	0	0	0	0	0	0	0	0	0	0	0	0	0	0	0
-dimethyl-,(E	0	0	8	1	0	3	3	0	0	5	2	0	0	0	0	0	0	0	0	0	0	0	0	0	0	0	0	0	0	0	0	0	0	0	0

)-	0	0	9	2	0	4	5	0	0	9	4	0	4	0	0	8	1	0	1	0	0	0	0	0	0	0	1	0	0	0	0	0	0	0	0
	0	0	0	9	0	8	8	0	0	0	5	0	7	0	0	3	5	0	9	0	0	0	0	0	0	0	4	0	0	0	0	0	0	0	0
	0.	0.	0.	0.	0.	0.	0.	0.	0.	0.	0.	0.	0.	0.	0.	0.	0.	0.	0.	0.	0.	0.	0.	0.	0.	0.	0.	0.	0.	0.	0.	0.	0.	0.	0.
3-Penten-2-o	0	0	0	0	0	0	0	0	0	0	0	0	0	0	0	0	0	0	0	0	0	0	0	0	0	0	0	0	0	0	0	0	0	0	0
ne	0	0	0	0	0	0	0	0	0	0	5	0	0	0	0	0	0	0	0	0	0	0	0	0	0	0	0	0	0	0	0	0	0	0	0
	0	0	0	0	0	0	0	0	0	0	4	0	0	0	0	0	0	0	0	0	0	0	0	0	0	0	0	0	0	0	0	0	0	0	0
	0	0	0	0	0	0	0	0	0	0	1	0	0	0	0	0	0	0	0	0	0	0	0	0	0	0	0	0	0	0	0	0	0	0	0
	0.	0.	0.	0.	0.	0.	0.	0.	0.	0.	0.	0.	0.	0.	0.	0.	0.	0.	0.	0.	0.	0.	0.	0.	0.	0.	0.	0.	0.	0.	0.	0.	0.	0.	0.
	0	0	0	1	0	2	0	0	0	0	0	0	2	0	0	0	0	0	2	0	0	0	0	0	0	1	0	0	0	0	0	0	0	0	0
Acetone	0	0	0	9	0	3	6	0	0	0	0	0	4	0	0	0	0	0	1	0	0	0	0	0	0	5	0	0	0	0	0	0	9	0	0
	0	0	0	3	0	3	5	0	0	0	0	0	5	0	0	0	0	0	6	0	0	0	0	0	0	6	0	0	0	0	0	0	8	0	0
	0	0	0	1	0	8	1	0	0	0	0	0	8	0	0	0	0	0	5	0	0	0	0	0	0	7	0	0	0	0	0	0	4	0	0
	0.	0.	0.	0.	0.	0.	0.	0.	0.	0.	0.	0.	0.	0.	0.	0.	0.	0.	0.	0.	0.	0.	0.	0.	0.	0.	0.	0.	0.	0.	0.	0.	0.	0.	0.
Hexane,3-met	0	0	0	0	0	0	0	0	0	0	0	0	0	0	0	0	0	0	0	0	0	0	0	0	0	0	0	0	0	0	0	0	0	0	0
hoxy-	0	0	0	0	0	0	0	0	0	0	0	0	0	0	0	2	0	0	0	0	0	0	1	0	0	0	0	0	0	0	0	0	0	0	0
	0	0	0	0	0	0	0	0	0	0	0	4	0	0	0	2	0	1	0	0	0	0	6	0	0	0	0	1	0	0	0	0	0	0	0
	0	0	0	0	0	0	0	0	0	0	0	1	0	0	0	4	0	1	0	0	0	0	8	0	0	0	0	0	0	0	0	0	0	0	0
	0.	0.	0.	0.	0.	0.	0.	0.	0.	0.	0.	0.	0.	0.	0.	0.	0.	0.	0.	0.	0.	0.	0.	0.	0.	0.	0.	0.	0.	0.	0.	0.	0.	0.	0.
2,4-Nonadien	0	1	0	0	0	0	0	0	0	0	0	0	0	0	0	0	0	0	0	0	0	0	0	0	0	0	0	0	0	0	0	0	0	0	0
e,(E,E)-	0	2	0	0	0	0	0	0	1	0	0	0	0	0	0	0	0	0	0	0	0	0	0	0	0	0	0	0	0	0	0	0	0	0	0
	5	1	0	0	0	0	0	4	4	0	0	0	0	0	3	4	0	0	0	0	0	0	0	0	0	0	0	0	0	0	0	0	0	0	0
	0	7	0	0	0	0	0	2	2	0	0	0	0	0	8	7	0	0	0	0	0	0	0	0	0	0	0	0	0	0	0	0	0	0	0
	0.	0.	0.	0.	0.	0.	0.	0.	0.	0.	0.	0.	0.	0.	0.	0.	0.	0.	0.	0.	0.	0.	0.	0.	0.	0.	0.	0.	0.	0.	0.	0.	0.	0.	0.
2-Ethyl-oxeta	0	0	0	0	0	0	0	0	0	0	0	0	0	0	0	0	0	0	0	0	0	0	0	0	0	0	0	0	0	0	0	0	0	0	0
ne	0	0	0	0	0	0	0	0	0	0	0	0	0	0	0	0	0	0	0	0	0	0	0	0	0	0	0	0	0	0	0	0	0	0	0
	3	0	0	0	0	0	0	0	0	0	0	0	0	0	0	0	1	0	0	0	0	0	0	1	0	0	0	0	0	0	0	0	0	0	0

	8	0	0	0	0	0	0	0	0	0	0	0	0	0	0	0	6	0	0	0	0	0	0	4	0	0	0	0	0	0	0	0	0	0	0
	0.	0.	0.	0.	0.	0.	0.	0.	0.	0.	0.	0.	0.	0.	0.	0.	0.	0.	0.	0.	0.	0.	0.	0.	0.	0.	0.	0.	0.	0.	0.	0.	0.	0.	0.
	0	4	9	0	0	0	0	0	0	2	0	0	0	4	0	0	0	0	0	0	0	0	0	0	0	0	0	0	0	0	0	0	0	0	0
Furan,2-ethyl	0	4	6	0	0	0	0	0	0	7	0	0	0	8	0	0	0	0	0	0	0	0	0	0	0	0	0	0	0	0	0	0	0	0	0
-	0	2	5	0	0	0	0	0	0	5	0	0	0	9	0	0	0	0	0	0	0	0	0	0	0	0	0	0	0	0	0	0	0	0	0
	0	0	4	0	0	0	0	0	0	9	0	0	0	4	0	0	0	0	0	0	0	0	0	0	0	0	0	0	0	0	0	0	0	0	0
	0.	0.	0.	0.	0.	0.	0.	0.	0.	0.	0.	0.	0.	0.	0.	0.	0.	0.	0.	0.	0.	0.	0.	0.	0.	0.	0.	0.	0.	0.	0.	0.	0.	0.	0.
	0	0	0	0	0	0	1	0	0	0	0	0	0	0	0	0	0	0	0	0	0	0	0	0	0	0	0	0	0	0	0	0	0	0	0
2-Butylfuran	0	1	0	0	0	0	1	0	0	0	0	0	0	0	5	0	0	0	0	0	0	0	0	0	0	0	0	0	0	0	0	0	0	0	0
	0	6	0	0	0	0	2	0	0	0	0	0	0	0	6	0	0	0	0	0	0	0	0	0	0	0	0	0	0	0	0	0	0	0	0
	0	3	0	0	0	0	5	0	0	0	0	0	0	0	4	0	0	0	0	0	0	0	0	0	0	0	0	0	0	0	0	0	0	0	0
	0.	0.	0.	0.	0.	0.	0.	0.	0.	0.	0.	0.	0.	0.	0.	0.	0.	0.	0.	0.	0.	0.	0.	0.	0.	0.	0.	0.	0.	0.	0.	0.	0.	0.	0.
	0	0	0	0	0	0	0	0	0	0	0	0	0	0	0	0	0	0	0	0	0	0	0	0	0	0	0	0	0	0	0	0	0	0	0
Furan,2-meth	0	0	0	0	0	0	1	0	0	0	0	0	0	0	0	0	0	0	0	0	0	0	0	0	0	0	7	0	0	0	0	0	0	2	2
yl-	0	0	0	0	0	0	4	0	0	0	0	0	0	0	0	0	0	0	0	0	0	0	0	0	0	0	3	0	0	0	0	0	0	4	4
	0	0	0	0	0	0	3	0	0	0	0	0	0	0	0	0	0	0	0	0	0	0	0	0	0	0	7	0	0	0	0	0	0	0	0
	0.	0.	0.	0.	0.	0.	0.	0.	0.	0.	0.	0.	0.	0.	0.	0.	0.	0.	0.	0.	0.	0.	0.	0.	0.	0.	0.	0.	0.	0.	0.	0.	0.	0.	0.
	0	0	0	0	0	0	0	0	0	0	0	0	0	0	0	0	0	0	0	0	0	0	0	0	0	0	0	0	0	0	0	0	0	0	0
Hexane,1,6-d	0	0	0	0	0	0	0	0	0	0	0	0	0	0	0	0	0	0	0	0	0	0	0	0	0	0	0	0	0	0	0	0	0	0	0
imethoxy-	0	0	0	0	0	0	0	0	0	0	0	0	0	0	0	0	0	7	0	0	0	0	0	0	0	0	0	0	0	0	0	0	0	0	0
	0	0	0	0	0	0	0	0	0	0	0	0	0	0	0	0	0	5	0	0	0	0	0	0	0	0	0	0	0	0	0	0	0	0	0
	0.	0.	0.	0.	0.	0.	0.	0.	0.	0.	0.	0.	0.	0.	0.	0.	0.	0.	0.	0.	0.	0.	0.	0.	0.	0.	0.	0.	0.	0.	0.	0.	0.	0.	0.
	0	0	0	0	0	0	0	0	0	0	0	0	0	0	0	0	0	0	0	0	0	0	0	0	0	0	0	0	0	0	0	0	0	0	0
Tetrahydrofur	0	0	0	0	0	0	0	0	0	0	0	0	0	0	0	0	0	0	0	0	0	0	0	0	0	0	0	0	0	0	0	0	0	0	0
an	0	0	0	0	0	9	2	0	0	0	0	4	3	0	0	0	0	0	2	0	0	0	0	0	0	0	0	0	0	0	0	0	0	0	0
	0	0	0	0	0	1	3	0	0	0	0	3	2	0	0	0	0	0	7	0	0	0	0	0	0	0	0	0	0	0	0	0	0	0	0

Furan,tetrahydro-3-methyl-	0.0000	0.0000	0.0000	0.0000	0.0000	0.0380	0.0000	0.0000	0.0000	0.0000	0.0000	0.0000	0.0415	0.0000	0.0000	0.0000	0.0000	0.0000	0.0000	0.0000	0.0000	0.0000	0.0000	0.0000	0.0000	0.0000	0.0000	0.0000	0.0000	0.0000	0.0000	0.0000	0.0000	0.0000	0.0000
Furan,2-pentyl-	6.3251	3.2140	15.6940	16.2128	15.7540	16.1324	14.7510	2.1190	1.3900	12.2640	14.2560	14.3310	11.1357	9.5933	0.0000	1.0980	2.2142	1.8775	3.6642	1.5684	0.0198	0.0000	1.9994	0.0000	0.0000	0.0000	0.0247	0.0000	0.0000	0.2573	0.0000	0.0000	0.0000	0.0000	0.0000
2,3-Pentanedione	0.0000	0.0000	0.0000	0.0090	0.0000	0.0000	0.0000	0.0000	0.0000	0.0000	0.0000	0.0000	0.0697	0.0000	0.0000	0.0000	0.0000	0.0000	0.0000	0.0000	0.0000	0.1253	0.3621	0.0000	0.0000	0.0000	0.0000	0.0697	0.2543	0.3324	0.0000	0.0000	0.0000	0.0000	1.2014
2,3-Hexanedione	0.0000	0.0000	0.0000	0.0101	0.0000	0.0000	0.0000	0.0000	0.0000	0.0000	0.0000	0.0000	0.0000	0.0000	0.0000	0.0000	0.0000	0.0000	0.0000	0.0000	0.0000	0.0000	0.0000	0.0000	0.0000	0.0000	0.0000	0.0000	0.0000	0.0000	0.0000	0.0000	0.0000	0.0000	0.0565
4,6-Octanedione	0.0000	0.0000	0.0000	0.0051	0.0000	0.0000	0.0000	0.0000	0.0000	0.0000	0.0000	0.0000	0.1057	0.0000	0.0000	0.0000	0.0000	0.0000	0.2350	0.0000	0.0000	0.0000	0.1057	0.0000	0.0000	0.9654	0.0000	0.0000	0.0000	0.5211	0.0215	0.0000	1.2213	0.0000	0.0000

	0.	0.	0.	0.	0.	0.	0.	0.	0.	0.	0.	0.	0.	0.	0.	0.	0.	0.	0.	0.	0.	0.	1.	0.	0.	0.	0.	0.	1.	1.	0.	0.	0.	0.	0.
	0	0	0	0	0	0	0	0	0	0	0	0	0	0	0	9	0	0	0	0	0	7	2	0	0	0	0	0	1	3	0	0	0	0	3
Decane	0	0	0	0	0	0	0	0	4	0	0	0	0	0	3	5	0	0	0	0	1	5	2	0	0	0	0	0	3	6	0	0	0	0	8
	0	0	0	0	0	0	0	0	8	0	0	0	0	0	6	4	0	0	0	0	5	1	5	0	0	0	0	0	6	5	0	0	0	0	6
	0	0	0	0	0	0	0	5	7	0	0	0	0	0	5	1	0	0	0	0	1	2	3	0	0	0	0	0	3	2	0	0	0	0	7
	0.	0.	0.	0.	0.	0.	0.	0.	0.	0.	0.	0.	0.	0.	0.	0.	0.	0.	0.	0.	0.	1.	0.	0.	0.	0.	0.	0.	1.	0.	0.	0.	0.	0.	0.
	0	0	0	0	0	0	0	0	0	0	0	0	0	0	2	0	0	0	0	0	0	0	0	0	2	0	0	0	2	0	0	4	0	1	0
Nonane	0	0	0	0	0	0	0	0	0	0	0	0	0	0	4	0	0	0	0	0	0	3	0	0	1	0	0	0	5	0	0	5	0	4	0
	0	0	0	0	0	0	0	0	0	0	0	0	0	0	1	0	0	1	0	0	0	6	0	0	5	0	3	0	3	0	0	2	0	2	0
	0	0	0	0	0	0	0	2	0	0	0	0	0	0	5	0	0	3	0	0	0	0	0	0	1	0	6	0	2	0	0	1	0	2	0
	0.	0.	0.	0.	0.	0.	0.	0.	0.	0.	0.	0.	0.	0.	0.	0.	0.	0.	0.	0.	0.	0.	0.	0.	0.	0.	0.	0.	0.	0.	0.	0.	0.	1.	0.
	0	0	0	0	0	0	0	0	0	0	0	0	0	0	0	0	0	0	0	0	0	0	2	0	0	0	0	2	0	3	1	1	0	1	3
Methylenechl	0	0	0	0	0	0	0	0	0	0	0	0	1	0	0	0	0	0	0	0	0	0	4	1	0	0	0	9	0	6	1	1	4	1	7
oride	0	0	0	0	0	0	0	0	0	0	0	0	4	0	0	0	0	0	0	0	0	0	1	4	2	3	1	3	0	5	5	2	2	4	7
	0	0	0	0	0	0	0	0	0	0	0	0	0	0	0	0	0	0	0	0	0	0	1	0	5	6	4	8	0	4	4	5	1	7	1
	0.	0.	0.	0.	0.	0.	0.	0.	0.	0.	0.	0.	0.	0.	0.	0.	0.	0.	0.	0.	2.	0.	0.	0.	0.	0.	0.	2.	0.	0.	0.	0.	0.	0.	2.
	0	0	0	0	0	0	6	0	0	0	0	0	0	0	0	0	0	0	0	0	3	0	1	0	0	0	0	1	0	5	2	0	0	0	6
Styrene	0	0	0	0	0	0	4	0	0	0	0	0	0	0	0	0	0	0	0	0	3	0	2	1	0	0	0	7	0	5	6	0	0	0	7
	0	0	0	0	0	0	2	0	0	0	0	0	0	0	0	0	0	0	0	0	5	0	2	1	0	0	0	8	0	2	5	0	0	0	8
	0	0	0	0	0	0	3	0	0	0	0	0	0	0	0	0	0	0	0	0	1	0	2	3	0	0	0	1	0	1	2	0	0	0	1
	0.	0.	0.	0.	0.	0.	0.	0.	0.	0.	0.	0.	2.	0.	0.	0.	0.	0.	0.	2.	0.	1.	2.	0.	0.	0.	2.	0.	1.	2.	2.	0.	0.	2.	0.
	0	0	0	0	0	0	0	0	0	0	0	0	6	0	5	6	0	0	1	6	1	3	3	7	2	6	6	0	0	6	0	6	9	4	3
Toluene	0	1	0	0	0	0	1	5	0	0	0	1	8	9	5	2	1	3	1	5	4	5	5	3	3	2	6	7	2	3	4	3	6	5	3
	2	0	7	0	1	6	1	9	8	8	2	5	5	8	2	1	3	5	2	2	0	2	2	7	5	4	0	8	1	5	8	2	5	1	2
	8	6	5	0	0	7	7	4	3	3	8	4	0	0	7	4	2	4	5	1	1	5	1	1	4	1	0	8	3	4	9	5	2	0	2
Benzene	0.	0.	0.	0.	0.	0.	0.	0.	0.	0.	0.	0.	0.	0.	0.	0.	0.	0.	0.	0.	0.	0.	0.	0.	0.	0.	0.	0.	0.	0.	0.	0.	1.	0.	0.

	0	0	0	0	0	0	0	0	0	1	0	0	0	0	4	0	3	0	0	0	0	7	0	0	0	7	0	0	7	0	0	0	2	0	0
	0	0	0	0	0	0	0	6	0	8	0	0	0	0	5	0	3	0	2	0	0	5	0	0	0	5	0	0	5	0	0	0	4	1	0
	0	0	0	0	0	0	0	1	0	3	0	0	0	0	8	0	9	0	1	0	0	6	0	0	0	2	0	0	4	0	0	0	3	1	0
	0	0	0	0	0	0	0	7	0	2	0	0	0	0	5	0	7	0	2	0	0	3	0	0	0	4	0	0	2	0	0	0	0	2	0
	0.	0.	0.	0.	0.	0.	0.	0.	0.	0.	0.	0.	0.	0.	0.	0.	0.	0.	0.	0.	0.	0.	0.	0.	0.	0.	0.	0.	0.	0.	0.	0.	0.	0.	0.
Benzene,1,3-dimethyl-	0	0	0	0	0	0	0	0	0	0	0	0	0	0	0	2	0	0	0	0	0	0	4	6	0	0	0	0	0	6	5	0	0	0	1
	0	0	0	0	0	0	0	0	1	0	0	0	6	0	0	2	0	0	0	0	0	0	2	3	0	0	0	5	0	3	2	2	0	0	4
	0	0	0	0	0	0	0	0	1	0	0	0	5	0	0	5	0	0	0	0	0	0	2	7	0	0	0	7	0	2	1	5	0	0	0
	0	0	0	0	0	0	0	0	9	0	0	0	0	0	0	5	0	0	0	0	0	0	2	2	0	0	0	1	0	1	3	4	0	0	1
